# The conformational flexibility of the C-terminus of histone H4 promotes histone octamer and nucleosome stability and yeast viability

**DOI:** 10.1186/1756-8935-5-5

**Published:** 2012-04-27

**Authors:** Myrriah S Chavez, Jean K Scorgie, Briana K Dennehey, Seth Noone, Jessica K Tyler, Mair EA Churchill

**Affiliations:** 1 Department of Biochemistry and Molecular Biology, University of Texas MD Anderson Cancer Center, Houston, TX, 77030, USA; 2 Department of Pharmacology and Structural Biology and Biophysics Program, University of Colorado, School of Medicine, 12801 East 17th Avenue, Aurora, CO, 80045-0511, USA

**Keywords:** Anti-silencing function 1 (Asf1), Histone H3/H4, Histone H4, Nucleosome, *Saccharomyces cerevisiae*

## Abstract

****Background**:**

The protein anti-silencing function 1 (Asf1) chaperones histones H3/H4 for assembly into nucleosomes every cell cycle as well as during DNA transcription and repair. Asf1 interacts directly with H4 through the C-terminal tail of H4, which itself interacts with the docking domain of H2A in the nucleosome. The structure of this region of the H4 C-terminus differs greatly in these two contexts.

****Results**:**

To investigate the functional consequence of this structural change in histone H4, we restricted the available conformations of the H4 C-terminus and analyzed its effect *in vitro* and *in vivo* in *Saccharomyces cerevisiae*. One such mutation, H4 G94P, had modest effects on the interaction between H4 and Asf1. However, in yeast, flexibility of the C-terminal tail of H4 has essential functions that extend beyond chromatin assembly and disassembly. The H4 G94P mutation resulted in severely sick yeast, although nucleosomes still formed *in vivo* albeit yielding diffuse micrococcal nuclease ladders. *In vitro*, H4G4P had modest effects on nucleosome stability, dramatically reduced histone octamer stability, and altered nucleosome sliding ability.

****Conclusions**:**

The functional consequences of altering the conformational flexibility in the C-terminal tail of H4 are severe. Interestingly, despite the detrimental effects of the histone H4 G94P mutant on viability, nucleosome formation was not markedly affected *in vivo*. However, histone octamer stability and nucleosome stability as well as nucleosome sliding ability were altered *in vitro*. These studies highlight an important role for correct interactions of the histone H4 C-terminal tail within the histone octamer and suggest that maintenance of a stable histone octamer *in vivo* is an essential feature of chromatin dynamics.

## **Background**

The nucleosome is the repeating unit of chromatin, which organizes all eukaryotic genomes [1]. As a complex of DNA with an octamer composed of two copies each of four histone proteins, the central histone H3/H4 tetramer wraps the central 80 base pairs (bp) of DNA, and the flanking H2A/H2B dimers wrap the remaining 67 bp of the DNA into a compact structure [2-4]. This structure is repressive to most, if not all, DNA dependent processes. For example, both nucleosome assembly and disassembly are critical for proper DNA replication, repair, and transcription [5,6]. These processes are regulated by a vast array of chromatin remodeling proteins, histone modification enzymes, and histone chaperones [7,8].

Histone chaperones are acidic proteins that facilitate histone deposition, exchange and eviction during nucleosome assembly and disassembly (reviewed in [5,9-11]). The histone chaperone, anti-silencing function 1 (Asf1), is highly conserved throughout eukaryotes [12-16] and binds to a heterodimer of H3/H4 [14,17]. Asf1 shields H3/H4 dimers from unfavorable DNA interactions and promotes the formation of favorable histone-DNA interactions [18]. Asf1 is also necessary for acetylation of H3 at lysine 56, which facilitates the assembly of newly synthesized H3/H4 into nucleosomes [19-22]. Whereas humans have two isoforms of Asf1, *Saccharomyces cerevisiae* has a single isoform, making it an excellent model system for structure-function analyses [13,16].

Crystal structures of Asf1 bound to the H3/H4 dimer show that Asf1 binds to H3 at the H3/H4 dimerization surface, physically blocking formation of the H3/H4 tetramer [23,24]. These structures also revealed that interactions with not only H3 but also H4 are required for Asf1 histone chaperone function [23,24]. In the Asf1-H3/H4 complex, the C terminus of H4 forms an antiparallel β sheet with the globular core of Asf1. However, the structure that H4 adopts in the nucleosome [3], requires a nearly 180 ° rotation about glycine 94 in order to form a parallel β sheet with H2A [3]. The structural dynamics of the H4 C-terminal tail, and its accessibility once H2A/H2B dimers are removed from the nucleosome, led to our suggestion that the H4 tail might facilitate chromatin disassembly/assembly via a ‘strand capture mechanism’ [23]. In this mechanism, Asf1 would capture the C-terminal tail of histone H4. This is important for the interaction of Asf1 with free H3/H4 dimers [23,24] and may also be relevant for the disassembly of H3/H4 dimers from chromatin. However, *in vitro*, Asf1 was unable to bind to the H3/H4 dimer in the context of the tetrasome [18], suggesting that other components are also required for tetrasome disassembly.

To investigate the functional consequence of altering the conformational flexibility in the C-terminal tail of H4, we analyzed the effects of restricting the range of motion available to the H4 C-terminus on Asf1-dependent activities by introducing the mutations G94A and G94P to the tail of H4 and studied their effect in yeast and *in vitro.* The G94A substitution was predicted to have little impact on the range of motion, whereas we anticipated that the G94P substitution would severely restrict H4 C-terminal tail flexibility. The structure and binding interactions of the mutant H4 G94P with Asf1 were similar to wild-type (WT) H4. However, yeast expressing only the G94P mutation were very sick, whereas yeast expressing only the G94A mutation grew like WT cells. Despite the detrimental effects of the G94P mutant on viability, nucleosome formation was not markedly altered *in vivo*. However, histone octamer stability and nucleosome stability as well as nucleosome sliding ability were reduced *in vitro*.

## **Results**

### **Restricting the conformational flexibility of the H4 C-terminal tail is detrimental to yeast viability**

**Figure 1:**
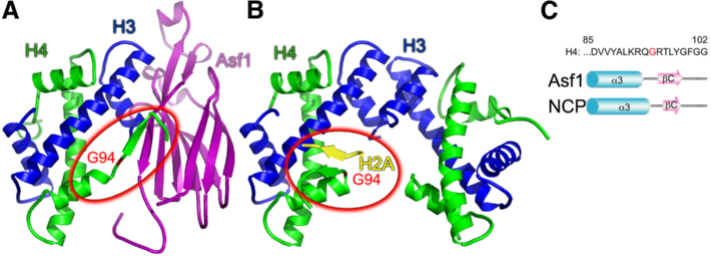
**Different conformations adopted by the H4 C-terminal tail within the nucleosome and when bound to anti-silencing function 1 (Asf1).** (**A**) Ribbon diagram showing the overall structure of the Asf1-H3/H4 complex (PDB:2HUE) [23], with Asf1 (violet), H3 (blue), and H4 (green). G94 is shown in red and the H4 C-terminal tail is circled in red. **(B)** Ribbon diagram showing the histone H3/H4 heterotetramer from the nucleosome core particle (PDB:1KX5) [26] oriented so that the H3/H4 dimer on the left is superimposed with the H3/H4 dimer in (A). Structures are colored as in (A) with H2A in yellow. **(C)** The amino acid sequence of the H4 C-terminal tail is shown with G94 in red aligned with the α3 helix and C-terminal β strand (βC) illustrated for H4 in the Asf1-H3/H4 complex and the nucleosome core particle (NCP), respectively.

There is an inherent conformational flexibility in the histone H4 C-terminal tail that is apparent from the distinct conformations that it adopts when in the nucleosome and when bound to Asf1 (Figure 1) [3,23,25]. This conformational change occurs at glycine 94. To restrict the conformational flexibility of the H4 C-terminal tail, we replaced G94 with either a proline, which is predicted to favor the more extended structure of the H4 C-terminus seen when H4 binds to Asf1, or an alanine, which is slightly more conformationally constrained than glycine and expected to have a smaller effect on C-terminal tail flexibility (Figure 1A). The mutant proteins and yeast strains will be referred to as H4^G94P^ and H4^G94A^, and H4G94P and H4G94A, respectively.

To examine the effects of these mutations *in vivo,* we used the previously characterized strain RMY102 [27], which has been used for other histone depletion studies [28-30]. RMY102 is deleted for the endogenous H3 and H4 genes (*HHT1HHF1HHT2* and *HHF2*) but contains plasmid pRM102, a *CEN* plasmid marked with *URA3* that bears histones H3 and H4 (*HHT2* and *HHF2*) under the control of the divergent *GAL1*/*GAL10* promoters (Tables 1 and 2). This plasmid allows RMY102 to maintain viability when grown on galactose containing medium. RMY102 was transformed with a second *CEN* plasmid marked with *TRP1* carrying WT H3 (*HHT2*) and either a WT or a mutant copy of H4 (*HHF2*), expressed under native promoter control. The new strains were cured of the *URA3* plasmid by growth on 5-fluoroorotic acid (5-FOA) leaving behind only the plasmid of interest.

**Table 1:** Plasmids used in this study

Plasmid	Characteristics	Source or reference
pFA6a-KANMX6		Longtine, 1998
pFA6a-his5 + MX6		Longtine, 1998
pRS314	*CEN6 ARSH4 TRP1*	Sikorski & Hieter, 1989
pRS414	*CEN6 ARSH4 TRP1*	Sikorski & Hieter, 1989
pRS316	*CEN6 ARSH4 URA3*	Sikorski & Hieter, 1989
pRM102	*CEN4 ARS1 p(GAL10)-HHT2 p(GAL1)-HHF2 URA3*	Mann & Grunstein, 1992
pEMHE81	pRS414 containing *HHT2* and *HHF2*	Hyland, 2005
pEMHE81H4G94P	pEMHE81 containing *HHT2* and *hhf2* G94P	This study
pEMHE81H4G94A	pEMHE81 containing *HHT2* and *hhf2* G94A	This study
pRS314-Asf1-Myc	pRS314 containing 13 × C-terminally Myc tagged Asf1	English, 2006
pST39T60xtal	Triple expression vector for Asf1-H3/H4	English, 2006
pST39T60 H4^G94P^	pST39T60xtal containing H4G94P mutation	This study
pET3a H4^T71C^	Containing histone H4 T71C mutation	Park, 2004
pET3a H4^T71C,^^G94P^	pET3a H4^T71C^ containing H4G94P mutation	This study
pET3a H4 ^T71C, ∆94^	pET3a H4^T71C^ containing H4truncated after 94	This study
pET-60-DEST	GST-His_6_-tag expression vector	Invitrogen
pET-60-yAsf1FL	*Saccharomyces cerevisiae* ASF1 inserted between the GST tag and the His_6_ tag coding sequences. The Asf1 -1 position (pro) was changed to (cys) for fluorophore attachment, and production of yAsf1*^532^	Donham, 2011

**Table 2:** Yeast strains used in this study

Strain	Genotype^a,b^	Parent, source or reference^c^
RMY102	*MATa ade2-101 his3∆200 lys2-801 trp1∆901 ura3-52 Thr-hht1 hhf1::LEU2 hht2 hhf2::HIS3* [pRM102]	Mann, 1992
SNY089	*MATa ade2-101 his3∆200 lys2-801 trp1∆901 ura3-52 Thr-hht1 hhf1::LEU2 hht2 hhf2::HIS3 asf1*::KANMX6 [pRM102]	RMY102
SNY093	*MATa ade2-101 his3∆200 lys2-801 trp1∆901 ura3-52 Thr-hht1 hhf1::LEU2 hht2 hhf2::HIS3 asf1*::KANMX6 [pEMHE81]	SNY089
SNY090	*MATa ade2-101 his3∆200 lys2-801 trp1∆901 ura3-52 Thr-hht1 hhf1::LEU2 hht2 hhf2::HIS3 ASF1*::13MycKANMX6 [pRM102]	RMY102
SNY091	*MATa ade2-101 his3∆200 lys2-801 trp1∆901 ura3-52 thr-hht1 hhf1::LEU2 hht2 hhf2::HIS3 ASF1*::13MycKANMX6 [pEMHE81]	SNY090
SNY092	*MATa ade2-101 his3∆200 lys2-801 trp1∆901 ura3-52 Thr-hht1 hhf1::LEU2 hht2 hhf2::HIS3 ASF1*::13MycKANMX6 [pEMHE81H4G94A]	SNY090
SNY095	*MATa ade2-101 his3∆200 lys2-801 trp1∆901 ura3-52 Thr-hht1 hhf1::LEU2 hht2 hhf2::HIS3 ASF1*::13MycKANMX6 [pEMHE81H4G94P]	SNY090
MCY073	*MATa ade2-101 his3∆200 lys2-801 trp1∆901 ura3-52 thr- hht1 hhf1::LEU2 hht2 hhf2::HIS3 ASF1*::13MycKANMX6 *RTT109::*6xGly-(FLAG)_3_::hphMX6 [pEMHE81]	SNY091
MCY074	*MATa ade2-101 his3∆200 lys2-801 trp1∆901 ura3-52 Thr-hht1 hhf1::LEU2 hht2 hhf2::HIS3 ASF1*::13MycKANMX6 *RTT109*::6xGly-(FLAG)_3_::hphMX6 [pEMHE81H4G94A]	SNY092
MCY075	*MATa ade2-101 his3∆200 lys2-801 trp1∆901 ura3-52 Thr-hht1 hhf1::LEU2 hht2 hhf2::HIS3 ASF1*::13MycKANMX6 *RTT109*::6xGly-(FLAG)_3_::hphMX6 [pEMHE81H4G94P]	SNY095
MCY076	*MATa ade2-101 his3∆200 lys2-801 trp1∆901 ura3-52 Thr- hht1 hhf1::LEU2 hht2 hhf2::HIS3 asf1*::KANMX6 *RTT109*::6xGly-(FLAG)_3_::hphMX6 [pEMHE81]	SNY093
W1588-4a	*MATα leu2-3,112 ade2-1 can1-100 his3-11,15 ura3-1 trp1-1*	Gift from R Rothstein
W1588-4c	*MATa leu2-3,112 ade2-1 can1-100 his3-11,15 ura3-1 trp1-1*	Gift from R Rothstein
BKD215	*MATα leu2-3,112 ade2-1 can1-100 his3-11,15 ura3-1 trp1-1 hht1 hhf1::*KANMX6	W1588-4a (see Methods)
BKD203	*MATa leu2-3,112 ade2-1 can1-100 his3-11,15 ura3-1 trp1-1 hht2 hhf2::HHT2 HHF2::TRP1*	W1588-4c (see Methods)
BKD204	*MATa leu2-3,112 ade2-1 can1-100 his3-11,15 ura3-1 trp1-1 hht2 hhf2::HHT2 HHF2::TRP1*	W1588-4c (see Methods)
BKD207	*MATa leu2-3,112 ade2-1 can1-100 his3-11,15 ura3-1 trp1-1 hht2 hhf2::HHT2 HHF2 G94P::TRP1*	W1588-4c (see Methods)
BKD210	*MATa leu2-3,112 ade2-1 can1-100 his3-11,15 ura3-1 trp1-1 hht2 hhf2::HHT2 HHF2 G94A::TRP1*	W1588-4c (see Methods)
MCY081	*MATa* /*MATα leu2-3,112/leu2-3,112 ade2-1/ade2-1 can1-100/can1-100 his3-11,15/his3-11,15 ura3-1/ura3-1 trp1-1/trp1-1 HHT2 HHF2::TRP1/HHT2 HHF2 HHT1 HHF1/hht1 hhf1::*KANMX6	BKD203 × BKD215
MCY084	*MATa* /*MATα leu2-3,112/leu2-3,112 ade2-1/ade2-1 can1-100/can1-100 his3-11,15/his3-11,15 ura3-1/ura3-1 trp1-1/trp1-1 HHT2 HHF2::TRP1/HHT2 HHF2 HHT1 HHF1/hht1 hhf1::*KANMX6 [pRM102]	MCY081
MCY091	*MATa leu2-3,112 ade2-1 can1-100 his3-11,15 ura3-1 HHT2 HHF2::TRP1 hht1 hhf1::*KANMX6 [pRM102]	Segregant from MCY081
MCY082	*MATa* /*MATα leu2-3,112/leu2-3,112 ade2-1/ade2-1 can1-100/can1-100 his3-11,15/his3-11,15 ura3-1/ura3-1 trp1-1/trp1-1 HHT2 hhf2G94A::TRP1/HHT2 HHF2 HHT1 HHF1/hht1 hhf1::*KANMX6	BKD210 × BKD215
MCY086	*MATa* /*MATα leu2-3,112/leu2-3,112 ade2-1/ade2-1 can1-100/can1-100 his3-11,15/his3-11,15 ura3-1/ura3-1 trp1-1/trp1-1HHT2 hhf2G94A::TRP1/HHT2 HHF2 HHT1 HHF1/hht1 hhf1::*KANMX6 [pRM102]	MCY082
MCY094	*MATa leu2-3,112 ade2-1 can1-100 his3-11,15 ura3-1 HHT2 hhf2G94A::TRP1 hht1 hhf1::*KANMX6 [pRM102]	Segregant from MCY082
MCY083	*MATa* /*MATα leu2-3,112/leu2-3,112 ade2-1/ade2-1 can1-100/can1-100 his3-11,15/his3-11,15 ura3-1/ura3-1 trp1-1/trp1-1 HHT2 hhf2G94P::TRP1/HHT2 HHF2 HHT1 HHF1/hht1 hhf1::*KANMX6	BKD207 × BKD215
MCY088	*MATa* /*MATα leu2-3,112/leu2-3,112 ade2-1/ade2-1 can1-100/can1-100 his3-11,15/his3-11,15 ura3-1/ura3-1 trp1-1/trp1-1 HHT2 hhf2G94P::TRP1/HHT2 HHF2 HHT1 HHF1/hht1 hhf1::*KANMX6 [pRM102]	MCY083
MCY097	*MATa leu2-3,112 ade2-1 can1-100 his3-11,15 ura3-1 HHT2 hhf2G94P::TRP1 hht1 hhf1::*KANMX6 [pRM102]	Segregant from MCY088
MCY021	*MATα leu2-3,112 ade2-1 can1-100 his3-11,15 ura3-1 trp1-1 hht1 hhf1::*KANMX6 *asf1::*his5 + MX6	BKD215
MCY043	*MATa leu2-3,112 ade2-1 can1-100 his3-11,15 ura3-1 trp1-1 asf1::*his5 + MX6	Segregant from BKD207 × MCY021

^a^Plasmids are indicated in square brackets.

^b^Strains derived from the same parent are grouped together.

^c^Unless noted, strains are from this study.

**Figure 2:**
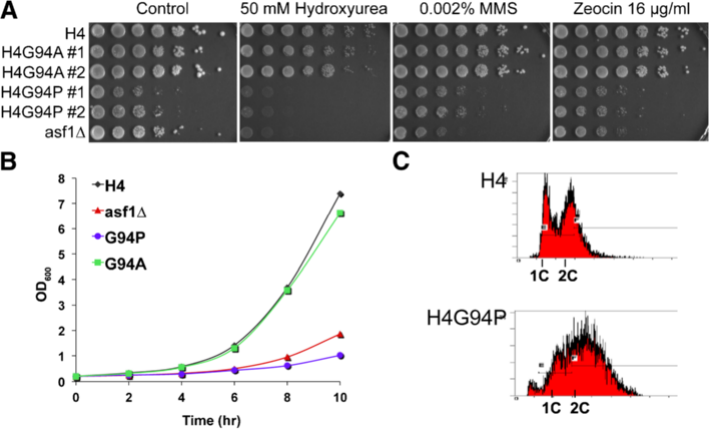
**Phenotypic consequences of limiting the conformational flexibility of the H4 C-terminal tail in yeast.** (**A**) Plasmid-borne expression of H4 mutants with limited C-terminal tail conformational flexibility leads to decreased colony formation and sensitivity to hydroxyurea (HU) in the RMY102 genetic background. Identical numbers of cells from strains SNY091 (wild-type (WT)), SNY092 (H4G94A), SNY095 (H4G94P) and SNY093 (*asf1∆*) were serially diluted (5 × dilutions) onto the indicated media, and photographed after 3 days. #1 and #2 indicate two independent isolates of the same strain. (**B**) Growth curves of the strains used in (A). (**C**) Cell cycle defects. Flow cytometry analysis of asynchronous cultures of the strains used above, stained with Sytox green.

As Asf1 is a histone H3-H4 chaperone, some Asf1 mutants that influence histone binding are sensitive to agents that induce replicative stress or DNA damage [23]. Therefore, we tested whether the H4G94P mutant, when present as the sole copy of histone H4, was sensitive to either replicative stress induced with hydroxyurea (HU), or DNA damage induced with either methyl methane sulfonate (MMS) or Zeocin. The H4G94A mutant was insensitive to these agents, whereas the H4G94P mutant was sensitive to HU, but not to MMS or Zeocin (Figure 2A). More striking, however, was the overall slow growth phenotype of yeast cells expressing H4^G94P^ relative to cells expressing either H4^WT^ or H4^G94A^ (Figure 2A, control plate), or to cells lacking Asf1 (*asf1∆*) (Figure 2A and B).

Growth defects can be due to slow cell cycle progression or accumulation of cells at one phase of the cell cycle. To determine whether the H4G94P mutant had normal cell cycle progression, we examined the DNA content of an asynchronous population of yeast cells by flow cytometry (Figure 2C). This method has been used in previous yeast histone mutant studies [31,32]. Compared to the WT H4 control, which shows defined 1 C and 2 C DNA content peaks, the cell cycle profile of the H4G94P mutant is highly disrupted and shifted rightward (Figure 2C). Treating cells with ethidium bromide to deplete them of mitochondrial DNA did not restore a normal cell cycle profile, indicating that the shift was not due to the accumulation of mitochondrial DNA (datanot shown). Taken together, these data show that in the RMY102 genetic background, the H4G94P mutation leads to growth defects more severe than, but distinct from, those caused by lack of Asf1.

To further analyze the H4 G94P and G94A mutations, and to avoid any plasmid copy number variation, we integrated the H4 G94A and G94P mutations into the W1588 genetic background (Table 2). Four haploid strains were constructed. In one, *HHT1* and *HHF1* were simultaneously replaced with a kanamycin resistance marker. In the other, *HHT2* and *HHF2* were replaced with a *TRP1* marked DNA segment containing both WT *HHT2* and either a mutant or WT copy of *HHF2* (see Methods). The strains were mated, sporulated, and subjected to tetrad analysis. As expected, both the TRP and KAN markers segregated 2:2, and approximately 25% of segregants from all crosses were, or were inferred to be, Trp + kanamycin resistant (Kan^r^). Viable Kan^r^ Trp+ segregants were obtained from the WT cross (100%) and the G94A cross (82%), while no viable Kan^r^ Trp+ segregants were recovered from the G94P cross. At the same time, kanamycin sensitive (Kan^s^) Trp+ segregants were viable, indicating that the G94P defect is due to a loss, not a gain of histone H4 function (Table 3).

**Table 3:** Viability of spores carrying H4G94P integrated into the genome

**Cross (relevant genotype)**	**Tetrads examined**	**Total segregants, viable + inviable**	**KAN**^**+**^**TRP**^**+**^**segregants, viable + inviable [inferred genotype]**	**kan**^**-**^**TRP**^**+**^**segregants**
BKD204 × BKD215*(HHT2 HHF2::TRP* x *hht1∆ hhf1∆::KAN*)	09	36 + 0	10 + 0 (100 %)	8 (22 %)
BKD210 × BKD215*(HHT2 hhf2*^*G95A*^*::TRP* x *hht1∆ hhf1∆::KAN)*	09	32 + 4	9 + [2] (82 %)	6 (17 %)
BKD207 × BKD215*(HHT2 hhf2*^*G95P*^*::TRP* x *hht1∆ hhf1∆::KAN)*	28	76 + 36	0 + [22] (0 %)	25 (22 %)

To rule out the possibility that the viability of the G94P mutant in the RMY102 genetic background, but not the W1588 genetic background, was due to the accumulation of a rare suppressor mutation, rather than inherent differences in the two strain backgrounds, we retransformed RMY102 with each of the WT and mutant H4 plasmids and tested their ability to grow on glucose and galactose containing medium as well as their ability to grow on 5-FOA containing medium (Additional file 1: Figure S1). Cells that a phenotypically Ura+, because they cannot lose the pRM012 plasmid, will die on 5-FOA, whereas those that have lost the plasmid, and are phenotypically Ura-, will survive. Given that 70% of the G94P expressing cells were viable on glucose and that nearly all colonies replica plated onto 5-FOA medium were viable, although slow growing, it seems very unlikely that the survival of the RMY102 cells is due to the accumulation of one or more suppressors following the introduction of the G94P mutant plasmid, but rather is due to inherent differences in the genetic backgrounds of the two different strains utilized. Notably, it is not unprecedented to find that the same histone mutation can be lethal in one background, yet viable in others [33,34].

**Figure 3:**
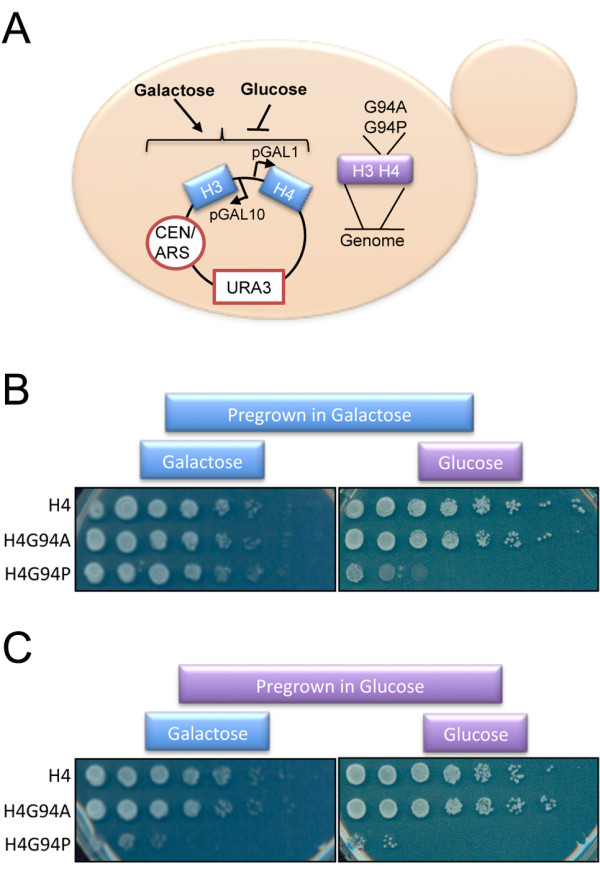
**The conformational flexibility of the H4 C-terminal tail is essential for growth.** (**A**) Illustration of the glucose shut-off strategy. Expression of plasmid borne wild-type (WT) H3/H4 under the control of the pGAL1/10 promoters in the W1588 genetic background is shut off by the addition of glucose leaving only the integrated copies of WT, G94A and G94P H4 expressed. (**B**) Integrated H4G94P forms colonies with low efficiency upon repressing expression of WT histones. Identical numbers of cells from strains MCY091 (H4), MCY094 (H4G94A), and MYC097 (H4G94P) were grown in galactose and serially diluted (5 × dilutions) onto plates containing glucose or galactose. (**C**) As in (B), but strains were grown in glucose for 5.5 h prior to plating. All media lacked uracil (−Ura) to maintain selection for the *URA3* marked plasmid expressing WT H3/H4. Plates were photographed after 3 days.

To further investigate this inviability, we used the four haploids described above to generate MCY091, 094 and 097 containing integrated H4, H4G94A and H4G94P marked with *TRP1*, respectively, as well as the pGAL histone plasmid, pRM102 [27] (Figure 3A). The derivationof these strains is detailed in Table 2. The strains were tested for viability by growing them in liquid cultures containing galactose and then plating equal numbers of cells onto plates containing either galactose, to maintain WT H4 expression, or glucose to repress WT H4 expression (Figure 3B). When galactose-grown cells were plated onto galactose medium, all strains grew equally well producing equivalent numbers of colonies. When galactose-grown cells were plated onto glucose medium, the WT and H4G94A strains formed similar numbers of colonies, but the H4G94P strain yielded roughly 1.5 × 10^4^-fold fewer colonies. This indicates that less than 0.01 % of the cells with integrated H4G94P survive to form a colony.

To determine whether cells could recover from H4G94P expression, we grew the cells in glucose medium for 5.5 h prior to plating them onto galactose and glucose plates (Figure 3C). Again, the H4G94P cells that were grown in glucose and plated onto glucose, formed approximately 1.5 × 10^4^-fold fewer colonies than the WT and H4G94A strains (Figure 3C). However, the H4G94P cells grown in glucose and then plated onto galactose, a condition that should restore WT H4 expression, formed approximately 625-fold fewer colonies than the WT H4 and H4G94A strains (Figure 3C). This shows that most cells cannot recover from even a short period with H4^G94P^ as the predominant version of H4 in the cell.

To determine how many times the H4G94P mutant cells can divide after repressing WT pGAL H4 expression, we grew WT, H4G94A and H4G94P cells in galactose liquid cultures and plated single cells onto glucose medium and counted how many times each cell had divided at given times (Additional file 1: Figure S2A). By 8 h most WT and H4G94A cells had divided three times, and five times by 16 h (Additional file 1: Figure S2B). In contrast, the H4G94P cells had divided only twice over 4 to 8 h, and stopped dividing by the third cell division (Additional file 1: Figure S2B). Similarly, in liquid cultures containing glucose we found that the H4G94P mutant cells divided approximately twice and stopped dividing after 5 to 6 h (Additional file 1: Figure S2C), while still increasing in size, as indicated by an increase in OD_600_ (Additional file 1: Figure S2E). These cultures were enriched in large budded cells (6 h) and both unbudded and large budded cells (8 h) at the expense of tiny, small, and medium budded cells compared to either H4G94A or WT cultures (Additional file 1: Figure S3A,D, Glu). DAPI staining did not show that the cells were arrested at any one point in the cell cycle (Additional file 1: Figure S3B,E, Glu), but a large percentage of H4G94P expressing cells counted across all budding categories displayed aberrant cell morphology (Additional file 1: Figure S3A,D,E,F).

It is important to note that even upon addition of glucose to repress transcription of the wild-type histones the repression might not be complete. However whatever WT histone expression remains cannot compensate for the loss of H4 function seen in the G94P mutant cells. This suggests that H4G94P, when present as the primary histone H4 species, is deleterious to cell viability in the W1588 genetic background and its effects are irreversible.

### Restricting the conformational flexibility of the H4 C-terminal tail distorts the structure of the Asf1-H3/H4 complex

Although the phenotype of the H4G94P mutant in yeast is more severe than the deletion of *ASF1* alone, given the importance of the C-terminal tail of H4 within the Asf1-H3/H4 structure [23,24], we were interested in whether the H4^G94P^ mutant protein altered the structure of the Asf1-H3/H4 complex. We determined the structure of Asf1 (1 to 169) bound to the H3/H4^G94P^ dimer at a resolution of 2.35 Å (Table 4; PDB ID 4EO5). The Asf1-H3/H4^G94P^ crystals formed in the same space group, P3_1_21 as the WT complex, which was used as a molecular replacement model (PDB:2HUE) [23] and similar crystal contacts occur in both structures. The unit cell dimensions differed by nearly 5%, which suggested that the H4^G94P^ substitution altered the Asf1-H3/H4 structure.

**Table 4:** Crystallographic data and refinement statistics for PBD ID 4EO5

**Statistic**	**Value**
Data collection statistics	
Space group	P3_1_21
Resolution (Å)	37.24 to 2.30 (2.38 to 2.30)
Unit cell dimensions (Å)	97.69, 97.69, 115.07
Observed reflections	147,637
Unique reflections	28,711
Completeness (%)	100 (100)
Redundancy	5.14 (5.18)
R_merge_^a^ (%)	9.0 (47.4)
< I/σ >	8.4 (2.4)
Refinement statistics	
Resolution range (Å)	15 to 2.35 (2.41 to 2.35)
R value (%)	20.4 (28.9)
Free R value (%)	24.2 (36.7)
Number of reflections used	25,471
Luzatti coordinate error (Å)	0.33
Average B factor main chain (Å^2^)	25.93
Root mean square deviation from ideality	
Bond angle (˚)	1.282
Bond length (Å)	0.013
Solvent atoms	169
Protein atoms	2844

Values in brackets represent those in the outermost shell.

^a^${\text{R}}_{\text{merge}}=\sum \left|I-<I>\right|/\sum I.$

**Figure 4:**
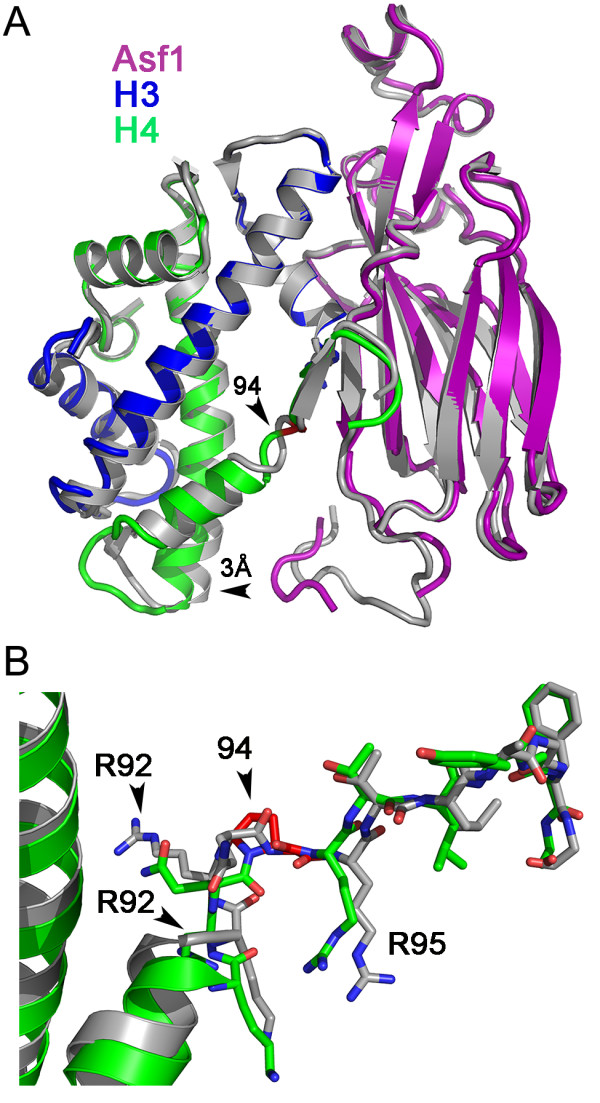
**Structural changes in the anti-silencing function 1 (Asf1)-H3/H4**^**G94P**^**complex.** (**A**) Superposition of the Asf1-H3/H4 and Asf1-H3/H4^G94P^ structures. Asf1-H3/H4 (PDB:2HUE) [23] (gray) is superimposed on the Asf1-H3/H4^G94P^ structure, colored as in Figure 1, with residue 94 in red. The direction of the 3-Å shift in the position of the histones in the Asf1-H3/H4^G94P^ structure is indicated by an arrow. (**B**) Structural alignment of the H4 C terminal tail in the Asf1-H3/H4^G94P^ (green) and Asf1-H3/H4 (gray) complexes. Nitrogen and oxygen atoms are colored blue and red, respectively.

Comparisons of the Asf1-H3/H4 and Asf1-H3/H4^G94P^ structures revealed both local and global effects conferred by the proline substitution. The root mean squared deviation (RMSD) for identical structures is approximately 0.4 Å based on the coordinate error of the two models. The RMSD between the Asf1-H3/H4 to the Asf1-H3/H4 ^G94P^ structures for all of the protein backbone atoms is 1.06 Å (Figure 4A). However, the RMSD values for the individual proteins are smaller, with the largest differences found in localized regions (Additional file 1: Table S1). The RMSD value for Asf1 overall is 0.94 Å, and this decreases to 0.47 Å when the Asf1 C-terminal tail (Asf1 146 to 164) is excluded from the calculation. The Asf1 C-terminal tail is shifted toward H4 and has a large RMSD of 1.58 Å, when compared to the WT complex. Histone H3 is nearly identical between the two structures with a backbone RMSD of 0.46 Å. Overall, histone H4 has an RMSD of 0.46 Å, but the RMSD of the C-terminal tail alone (H4 92 to 101) is 1.35 Å, whereas the core of the protein excluding the tail has an RMSD of 0.29 Å. Although these local differences are small, their effects are additive and result in a scissoring apart of the histones from Asf1 in the vicinity of the Asf1 C-terminal region by nearly 3 Å.

The G94P substitution causes structural differences near the interface of Asf1 and H4. Close inspection of the site of the G94P substitution (Figure 4B) shows an unraveling of the last turn of the third α helix of H4 and a reorientation of Arg92. In the Asf1-H3/H4^G94P^ complex, Arg92 rotates nearly 70 degrees and 8 Å to interact with residues Asp160 and Asp162 of the Asf1 C terminal tail. This increases the distance between the globular core of Asf1 and the histones, while at the same time bringing the Asf1 C-terminus toward H4. These give rise to a significant structural change in the Asf1-H4 interface of the complex. In contrast, the interaction of Asf1 with H3 is only slightly altered in the Asf1-H3/H4^G94P^ mutant complex. Thus, the overall structure of the Asf1-H3/H4^G94P^ complex differs from the WT complex, and this is largely due to the changes in the H4 C-terminus from the inability of the proline to adopt the same conformation as Gly94 of H4.

### Asf1 binds to histones with H4^G94P^*in vitro* and *in vivo*

**Figure 5:**
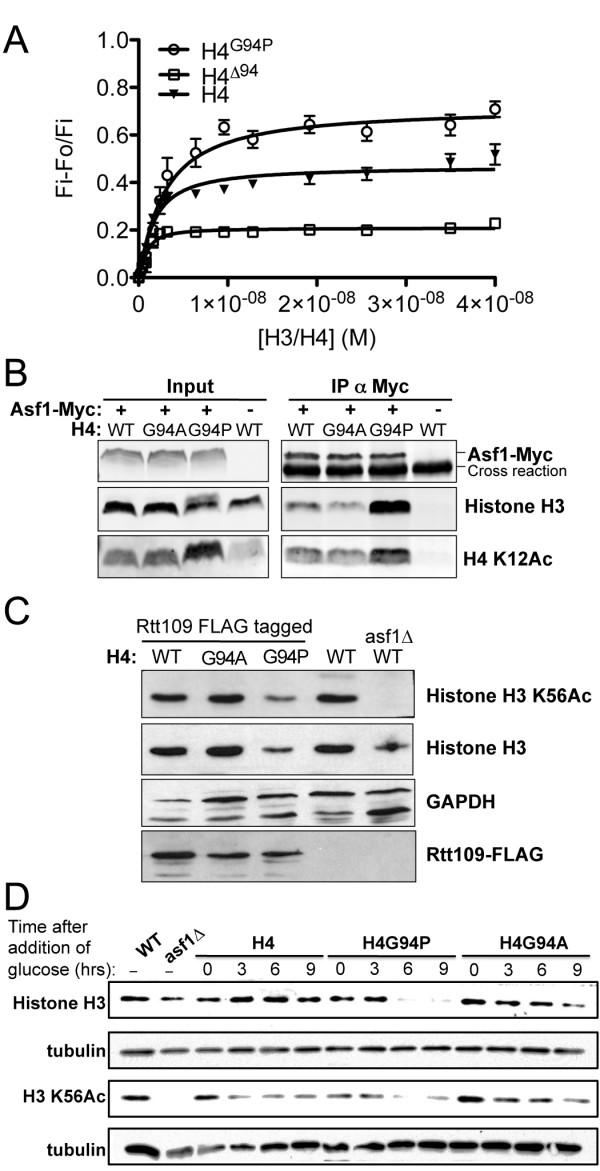
**Anti-silencing function 1 (Asf1) binds to H3/H4**^**G94P**^**and H3/H4 lacking the C-terminal tail*in vitro*and*in vivo.*** (**A**) H3/H4^G94P^ (○), H3/H4^∆94^ (□), and H3/H4 (▼) binding to 1 nM yAsf1*^532^ observed by fluorescence-quenching titration. The data were fitted with a ligand-depleted binding model (Equation 1). **(B)** H4^G94P^ coimmunoprecipitates with Asf1 more effectively than wild-type (WT) H4. Strains SNY091 (WT with Asf1-Myc), SNY092 (G94A with Asf1-Myc), SNY095 (G94P with Asf1-Myc), and RMY102 (WT without Asf1-Myc) were subject to anti-Myc immunoprecipitation, followed by western blotting for H3 or H4 (using antibody against H4 acetylated on lysine 12 (H4 K12Ac)). **(C)** Reduced amounts of H3 K56Ac in the H4G94P mutant is due to reduced overall histone levels, not reduced Rtt109 levels. Equivalent amounts of total protein extracted from strains MYC073 (WT with Rtt109-FLAG), MCY074 (G94A with Rtt109-FLAG), MCY075 (G94P with Rtt109-FLAG), RMY102 (WT) and SNY093 (WT asf1∆) were analyzed by western blotting for the indicated proteins. **(D)** The loss of total H3 is apparent upon repression of WT histone expression in strains carrying an integrated copy of the gene encoding H4G94P. Total protein extracts of strains W1588-4a (WT), MCY043 (asf1∆), MCY091 (H4), MCY094 (H4G94A), and MYC097 (H4G94P) were made at the indicated times after addition of glucose and western blotted for the indicated proteins. The same DNA equivalents (10 μg DNA) were loaded in each lane.

With differences between the WT and H4^G94P^ interactions with Asf1, we assessed whether the mutation wouldprevent Asf1 from binding to the histones with a normal affinity. Previously, we found a tight association between full-length yeast Asf1 and the H3/H4 dimer using a fluorescence-quenching assay [18]. Using the same approach, we analyzed the binding of Asf1 to H3/H4^G94P^. Unlabeled H3/H4^G94P^ dimers quench the Alexa Fluor 532 signal of labeled yAsf1*^532^, and the H3/H4^G94P^ mutant shows a slight increase in fluorescence quenching compared to the WT H3/H4 (Figure 5A). These data were fitted with a ligand depletion binding model (Equation 1) and gave a K_D_ of 2.42 ± 0.1 nM. This was almost identical to the K_D_ of 2.5 ± 0.7 nM measured for the WT H3/H4 to Asf1 [18], and showed that the H4^G94P^ mutation did not alter binding to Asf1 *in vitro*.

To complement the *in vitro* binding analyses, we took advantage of the fact that the G94P mutant, while sick, was viable in the RMY102 genetic background. This allowed us to examine the interaction between Asf1 and H3/H4 ^G94P^ mutant dimers by coimmunoprecipitation analysis in a strain that expressed only the mutant histones. H3/H4 coimmunoprecipited with Myc-tagged Asf1 to a similar degree in both the WT and G94A mutant strains (Figure 5B). However, the H3/H4^G94P^ mutant coimmunoprecipitated with Asf1 much more effectively (Figure 5B). These data show that while the affinity of Asf1 for H4 is not altered by the H4G94P mutation *in vitro*, in the cell, H3/H4^G94P^ is more likely than WT H3/H4 to accumulate in a complex with Asf1.

A possible explanation for the increased abundance of H4^G94P^ in Asf1 complexes *in vivo* could be that the G94P mutation reduces the affinity of H3/H4 for other histone chaperones. These chaperones, such as CAF-1 and Rtt106, normally deposit histones onto DNA [7] and this function is enhanced by acetylation of histone H3 on K56 (H3 K56Ac) [35]. Therefore, we examined the levels of total H3 K56Ac (Figure 5C) and found reduced levels in cells expressing H3/H4^G94P^. The level of Rtt109, the sole H3K56 acetyltransferase in yeast, was unchanged in cells expressing H3/H4^G94P^ (Figure 5C), and surprisingly, the decrease in H3 K56Ac levels in the H4G94P mutant actually paralleled a decrease in the total amount of histone H3 (Figure 5C). The decreased amount of H3 and H3 K56Ac was exacerbated upon repression of the WT histones (Figure 5D). We also found that the total level of H4 protein in the H4G94P mutant is reduced (see below). As such, the reduced level of H3 K56Ac in the H4G94P mutant is probably related to the reduced total amount of H3/H4 rather than a defect in the acetylation reaction *per se.* Given the reduced levels of H3/H4 in the H4G94P mutant, the increase in H3/H4 bound to Asf1 is even more striking (Figure 5B).

### The H4 tail, but not its conformational flexibility, is important for Asf1 to dissociate H3/H4 tetramers into H3/H4 dimers *in vitro*

Although both H3 and H4 bind to Asf1, the contribution of each to the overall binding affinity has not been measured. We analyzed the binding of H3/H4 dimers with a C-terminal truncation of H4 at amino acid 94 (H4^∆94^), using a fluorescence-quenching assay to obtain the K_D_ (Figure 5A). The binding affinity of the H3/H4^∆94^ dimer to yAsf1*^532^ was 0.4 ± 0.1 nM, which is also similar to the binding of Asf1 to the WT H3/H4. However, the degree of Asf1 quenching was much lower than observed for the WT histones, which suggests that the mode of Asf1 binding may be slightly different than for H3/H4 or H3/H4^G94P^.

The increased amount of H3/H4 bound to Asf1 in the G94P mutant might reflect an enhanced ability of Asf1 to disassemble H3/H4^G94P^ from the DNA compared to WT H3/H4. We attempted to measure the effect of these mutants on chromatin disassembly in yeast at the *PHO5* promoter [36], but the mutant cells were too sick to succeed with this assay. However, electrophoretic mobility shift assays (EMSA), showed that similar to WT tetrasomes [18], tetrasomes formed with H3/H4^G94P^ or H3/H4^∆94^ could not be dissociated in the presence of Asf1 *in vitro* (Additional file 1: Figure S 4). This result indicates that H3/H4 bind to DNA more tightly than they bind to Asf1 *in vitro*. For Asf1 to be able to remove histones from the DNA within the cell, histone modifications or additional proteins will be required [18].

**Figure 6:**
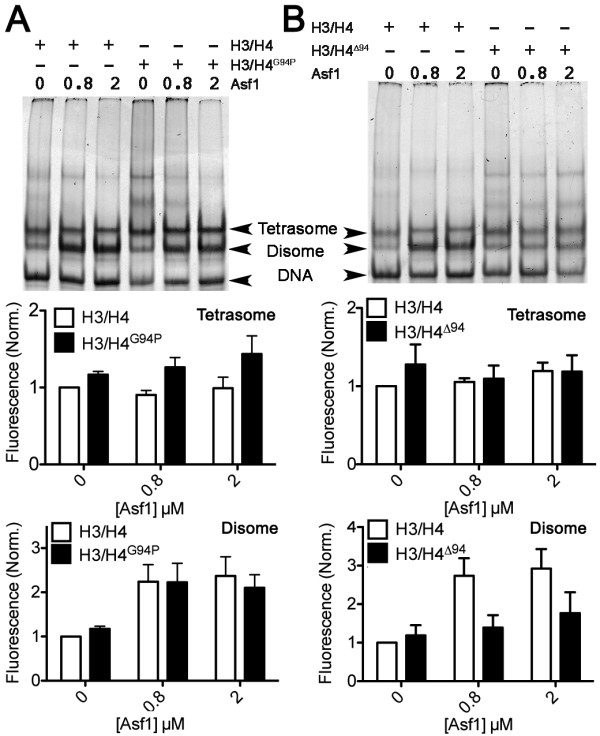
**Lack of the H4 C-terminal tail but not H4**^**G94P**^**alters anti-silencing function 1 (Asf1)-mediated disome formation.** H3/H4^G94P^**(A)** or H3/H4^∆94^**(B)** were compared to wild-type (WT) H3/H4, all at 0.8 μM dimer concentration, for their ability to form tetrasomes and disomes on 80 bp 5 S DNA (0.4 μM) in the absence and presence of Asf1 (0, 0.8, 2.0 μM). Upper panels show images of SYBR Green I stained DNA, and lower panels show the quantitation of the amount of disomes and tetrasomes formed for each type of histone, from at least three independent experiments. Tetrasome and disome levels were normalized to the WT H3/H4 sample in the absence of Asf1.

Previously, we showed that Asf1 helps form DNA-H3/H4 complexes [18]. To determine whether the C-terminal tail of H4 influences the ability of Asf1 to aid the assembly H3/H4-DNA complexes, we examined the formation of disomes (H3/H4-DNA) and tetrasomes ((H3/H4)_2_-DNA) by EMSA. H3/H4^G94P^, H3/H4^∆94^, H3/H4 proteins were incubated in separate reactions with Asf1 before adding DNA. Figure 6A shows that the H4^G94P^ mutation does not alter the assembly of disomes or tetrasomes relative to that observed previously for the WT H3/H4. The deletion of the H4 C-terminal tail has little effect on tetrasome formation, but disome formation is greatly decreased compared to WT H3/H4 (Figure 6B and [18]). This decrease likely reflects the lower amount of Asf1-H3/H4 complex available due to the inability of Asf1 to dissociate H3/H4 tetramers that lack H4 C terminal tails. This result shows that the H4 C-terminal tail is needed for Asf1 to dissociate H3/H4 tetramers and produce H3/H4 dimers that are competent to form disomes.

### Limiting the flexibility of the C-terminal tail of H4 yields diffuse nucleosomal ladders in yeast

**Figure 7:**
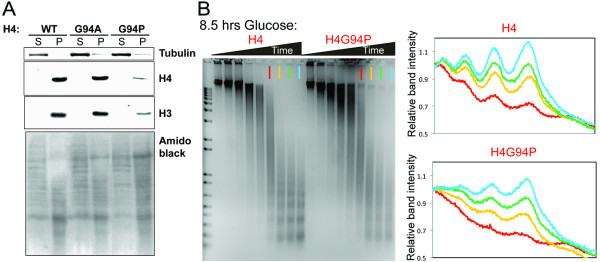
**Analysis of chromatin structure from yeast with the H4G94P mutation.** (**A**) Chromatin fractionation showing reduced amounts of histones on H4G94P genomes. Total protein extracts from equivalent numbers of cells from strains MCY073 (wild-type (WT)), MYC074 (G94A) and MCY075 (G94P) were fractionated into supernatant (S) containing soluble proteins and free histones and pellet (P) containing chromatinized proteins. Tubulin is a marker for effective separation of the supernatant and pellet fractions. Amido black staining of the membrane used for the western blots is shown below. (**B**) Micrococcal nuclease (MNase) accessibility is not increased in H4G94P mutants but the ladders become diffuse. Left panel, ethidium bromide stained gel of MNase treated chromatin from strains MCY091 (WT) and MCY097 (G94P) 8.5 h after repressing WT histone H4 expression (see Figure 3A). Right panel, densitometry traces emphasize the diffuse ladders of the H4G94P mutant.

The inviability of the H4G94P mutant could be a consequence of altered chromatin structure, which we examined by cell fractionation, micrococcal nuclease (MNase) accessibility and chromatin immunoprecipitation (ChIP) experiments. The total levels of H3 are reduced in the H4G94P mutant (Figure 5D), leading us to ask whether this reflects a loss of histones from the genome or a loss of free histones. We separated free and chromatin bound histones and found that the loss of H3 and H4 in the H4G94P mutant correlates with loss of histones from chromatin (Figure 7A). To determine whether nucleosome occupancy on the genome is reduced in the H4G94P mutant we used MNase. The MNase accessibility and quality of the nucleosomal ladders was identical for WT and H4G94P mutants when WT H4 was expressed (Additional file 1: Figure S5). Likewise, after repressing WT H4 expression for 8.5 h in glucose the extent of MNase accessibility was not notably altered in the H4G94P mutant, although the nucleosomal ladders became more diffuse (Figure 7B).

Given the reduced levels of H3 and H4 that we consistently observed by western blotting in the H4G94P mutant, it was surprising that the H4G94P mutant did not have increased accessibility to MNase. Therefore, we performed ChIP analyses of both H3 and H2B at a variety of locations throughout the yeast genome. In agreement with the MNase assays, these analyses showed no significantly decreased histone occupancy on chromatin in the H4G94P mutants (Additional file 1: Figure S 6).

### The H4 C-terminal tail and its conformational flexibility is important for the formation of stable histone octamers *in vitro*

**Figure 8:**
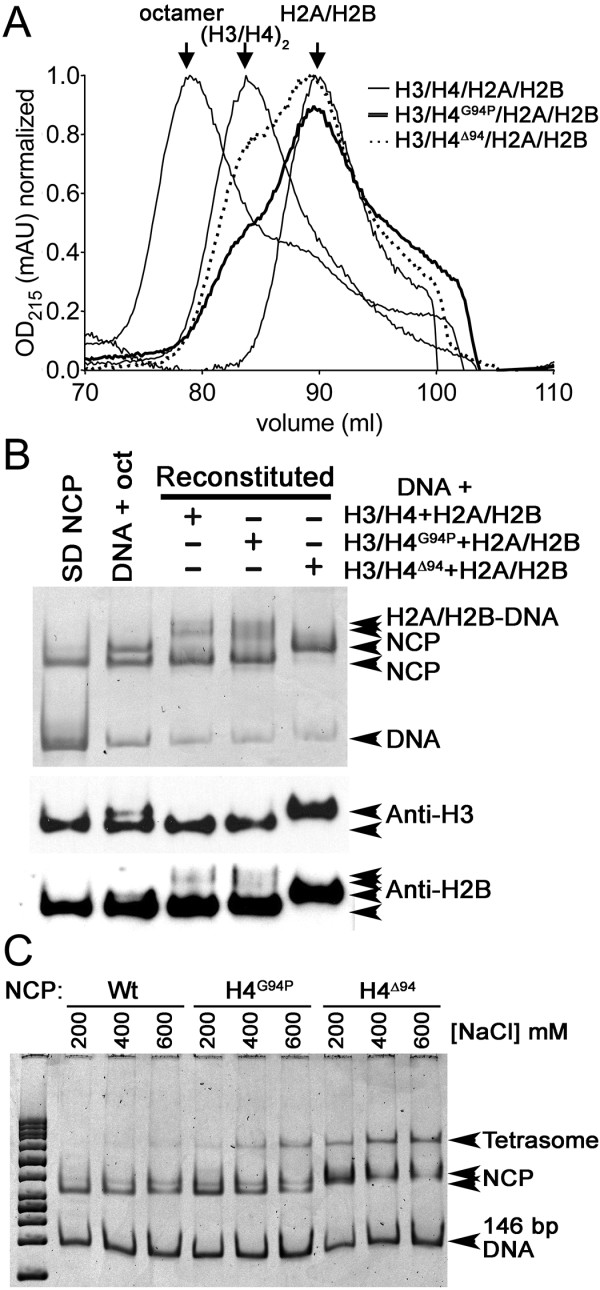
**Effects of H4 tail mutations on histone octamer and nucleosome formation.** Size exclusion chromatography (SEC) profile of histone octamer species shows that H4^G94P^ and H4^∆94^ fail to form octamers. The wild-type (WT) elution positions of each species of WT H3/H4 octamer, H3/H4 tetramer, and H2A/H2B dimers are shown as thin lines. The samples containing H4^G94P^ (dark line) and H4^∆94^ (dotted line), prepared identically to the WT octamer, elute at the tetramer and dimer elution volumes. (**B**) H4^G94P^ and H4^∆94^ form nucleosome core particles (NCPs). WT NCPs were made with 146 bp 601 DNA and histones using salt dialysis (SD NCP), direct addition microscale reconstitution (DNA + oct), and microscale reconstitution (DNA + H3/H4 + H2A/H2B) procedures. H4^G94P^ and H4^∆94^ containing NCPs were formed using the microscale reconstitution (DNA + H3/H4 + H2A/H2B) procedure. Arrows indicate the positions of the WT and aberrant NCPs [37]. Lower panels are western blots made from the same gel with the indicated antibodies. (**C**) Electrophoretic analysis of NCP stability. NCPs formed by the microscale reconstitution (DNA + H3/H4 + H2A/H2B) procedure with WT histones, H4^G94P^, or H4^∆94^, were incubated with 200, 400, or 600 mM NaCl in the buffers for 1 h prior to electrophoresis. Arrows indicate the positions of the NCPs, H2A/H2B-DNA complexes, H3/H4-DNA complexes (tetrasomes), and free DNA.

Given that the C-terminal region of H4 contacts H2A within the nucleosome, we investigated whether decreasing the flexibility of the C-terminal tail would alter the formation of histone octamers *in vitro*. Three distinct histone octamers were prepared [37] and analyzed by size-exclusion chromatography; all included H2A, H2B and H3, but H4 was varied with WT H4, H4^G94P^, and H4^∆94^. Both samples, containing H4^∆94^ and H4^G94P^, had elution profiles that resembled a combination of H3/H4 tetramers and H2A/H2B dimers, rather than octamers (Figure 8A). The presence of all four full-length proteins was confirmed by both SDS-PAGE (Additional file 1: Figure S7A) and MALDI-TOF mass spectrometry (Additional file 1: Figure S7B). Both the G94P mutation and C terminal deletion reduce the ability of the histones to assemble into stable octamers, which highlights the importance of the presence and correct structure or dynamics of the C-terminal tail of H4 in octamer formation. Given this result, we wondered whether overexpression of histone H2A/H2B in yeast might partially compensate for the altered conformational freedom of the H4G94P mutant. However, overexpression of H2A/H2B from a high copy plasmid did not restore normal growth to the G94P mutant (data not shown).

### The H4 C-terminal tail and its conformational flexibility is required for nucleosome stability and efficient nucleosome remodeling *in vitro*

Next, we assessed the ability of the mutant H4 proteins to assemble nucleosome core particles (NCPs). The 146 bp, strong nucleosome positioning DNA fragment, ‘601’ [38], was used to assemble NCPs from H2A, H2B, H3, and H4^WT^, H4^∆94^ or H4^G94P^ using several different approaches. NCPs formed by WT octamers and DNA migrate as a single band (Additional file 1: Figure S8A,B). However, the WT NCPs reconstituted as a combination of tetramers, dimers, and DNA migrate as two bands. The NCP formed with H4^G94P^ is similar to the NCP formed from H3/H4 tetramers, H2A/H2B dimers, and the 601 DNA fragment. However, the migration of the H4^∆94^ NCP is slower than those containing H3/H4 and H3/H4^G94P^. Western blot analyses show the presence of both H3 and H2B in the NCPs formed by each species of H4 (Figure 8B). The migration profile of the H4^∆94^ NCP resembles NCPs containing histone H2A C-terminal truncation mutants [39], which have been interpreted to be due to an open NCP structure [39,40].

Nucleosome stability is related to the tolerance of the particle to increased heat and ionic strength [41]. In order to compare the relative stability of the three NCPs, the ionic strength of the solution was increased and the resulting species analyzed by EMSA (Additional file 1: Figure S8B,C). Although WT NCPs resisted dissociation of their H2A/H2B dimers, as evidenced by the overall lack of tetrasomes seen by EMSA at salt concentrations up to 600 mM, both of the mutant NCPs dissociated into tetrasomes at salt concentrations of only 400 mM or less. Similar results were obtained by heat-induced dissociation of the NCPs (data not shown). These results, combined with the size exclusion data, indicate that the H4^G94P^ and H4^∆94^ mutants fail to form stable octamers, and although they do form NCPs, these are less stable to ionic strength than WT NCPs. The mobility of the H4^G94P^ NCP is more similar to the WT NCP than it is to the H4^∆94^ NCP, which is clearly altered in its structure and appears to form an open NCP structure, similar to that observed with deletion of the C-terminal tail of H2A [39].

**Figure 9:**
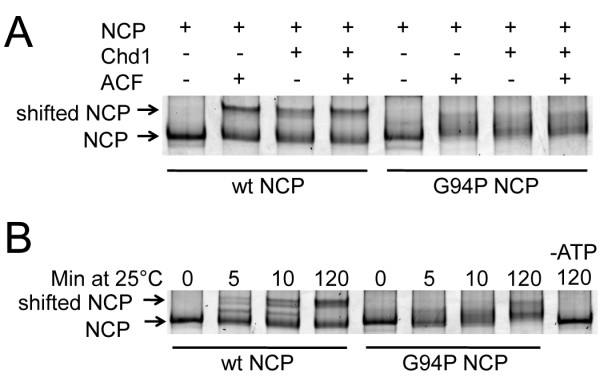
**H4G94P nucleosomes are less effectively remodeled by chromodomain-helicase-DNA-binding protein 1 (Chd1) or d**-**ATP-utilizing chromatin assembly and remodeling factor (ACF) than wild-type (WT) nucleosomes.** (**A**) 12 nM nucleosome core particles (NCPs) were incubated with 5 nM dACF and/or 1 nM Chd1 for 120 minutes. Wild-type nucleosomes are shifted equally well in the presence of dACF or Chd1, or the two proteins in combination. G94P nucleosomes cannot be fully shifted by either of the proteins or the combination. (**B**) 12 nM NCPs were incubated with 1 nM Chd1 for the stated time points. Chd1 fully slides 50 % of the WT NCP after 120 minutes. Only a small percentage of the G94P NCP is fully shifted after 120 minutes. Far right lane, WT NCP reaction in the absence of ATP.

The diffuse nucleosomal ladders that were observed in the MNase cleavage experiments (Figure 7B) suggested that some aspect of histone removal, assembly, or remodeling of nucleosomes may be altered by the H4G94P mutant. Therefore, we tested whether NCPs positioned asymmetrically on a 208 bp template were still substrates for nucleosome remodeling enzymes. Chromodomain-helicase-DNA-binding protein 1 (Chd1) and ATP-utilizing chromatin assembly and remodeling factor (ACF) are ATP dependent remodelers that are capable of moving nucleosomes from such an off-centered to a centered position on the DNA as assessed by EMSA. Figure 9A shows that dACF and Chd1 shift WT nucleosomes much more efficiently than G94P-containing nucleosomes [42-44]. A time course of the remodeling reaction with Chd1 shows rapid remodeling for the WT and much slower and less complete remodeling for the G94P NCPs (Figure 9B).

## Discussion

Due to the intrinsic conformational flexibility of glycine, the C-terminal tail of histone H4 is predicted to have a wide range of orientations. Substitution of the glycine with alanine restricts this flexibility somewhat, while substitution with proline limits this conformational freedom even further. The effects of this change in flexibility *in vivo* are dramatic. Although the H4G94A mutation is well tolerated by yeast, the H4G94P mutation is not, and leads to an altered cell cycle profile and inviability after two or three cell divisions (Figures 2 and 3).

The G94P H4 is compatible with the formation of an Asf1-H3/H4 complex. The Gly 94 phi and psi angles, of −52.5 ° and 145 °, in the complex of H3/H4^G94P^, differ from those in the WT Asf1-H3/H4 complex (75.4 ° and −175 °) [23], but the Asf1-H3/H4^G94P^ structure was only slightly distorted compared to the WT (Figure 4). Although the binding affinity was not altered by this H4 substitution *in vitro,* Asf1 sequesters H4^G94P^ to a greater degree than WT H4 *in vivo* (Figure 5). If sequestration of the histone by a chaperone was the origin of the mutant phenotype, then deletion of the gene encoding the chaperone should rescue the mutant, as was previously shown for the histone chaperone Rtt106 and other histone H4 mutants [32]. However, neither deletion nor overexpression of *ASF1* improved the growth of the G94P mutant (data not shown). Further, if the reason for the H4G94P mutant phenotype is due to histone sequestration by Asf1 or other chaperones, the mutant should be dominant over WT H4, which it is not.

Unexpectedly, we found reduced total histone levels in the H4G94P mutant, along with minor alterations in the MNase accessibility and genomic occupancy of histones. These results were obtained consistently with two sets of strains from two different genetic backgrounds. Possible explanations for these observations include the unlikely possibility that there is differential specificity of the H3 antibody in western blotting and ChIP analyses. Alternatively, it is possible that only a subset of histones, perhaps those that are tightly bound within nucleosomes, are monitored by the MNase accessibility and ChIP analyses, and that there is a looser subset of histones that are invisible to these assays that are absent in the H4G94P mutant. Precedent for this idea is provided by mutants of Asf1 and the Rtt109 histone acetyl transferase, which are more resistant to MNase digestion than WT cells [45,46] and have a higher histone occupancy by ChIP analysis (data not shown). However, over half of the histones in an *asf1* mutant are either not chromatin associated or are easily extracted from DNA in low amounts of detergent [47]. *In vitro,* nucleosome stability was reduced for NCPs containing H3/H4^G94P^, while in yeast, H3/H4 occupancy on the DNA as determined by ChIP and MNase accessibility was not significantly reduced in the H4G94P mutant. This discrepancy is likely due to the formaldehyde fixation step preceding the MNase and ChIP assays, which likely retains the structure of the nucleosomes.

Why is the H4G94P mutant inviable in yeast? This could be due to the substantially decreased levels of histones in this mutant. It is possible that the H4G94P mutation renders the histones more likely to be degraded due to having an altered conformation. If the H4^G94P^ protein impedes chromatin assembly, it may lead to an increase in free histones that are subsequently degraded via a Rad53-dependent mechanism following DNA replication [48]. However, we are not convinced that an inadequate supply of histones explains the inviability of the H4G94P mutant, because additional H4^G94P^ supplied from a high copy plasmid, did not improve viability or growth compared to additional H4^G94P^ supplied from a low copy plasmid (data not shown).

Although the steady state of chromatin structure in the H4G94P mutant does not appear to be greatly disrupted *per se* (from the MNase and ChIP analysis), it is possible that the rates of histone removal and assembly are altered in the H4G94P mutant *in vivo*. This could be due to reduced amounts of histones feeding into the chromatin assembly pathway because they remain bound to other histone chaperones. Alternatively, the conformational flexibility in the H4 C-terminus might be important for the disassembly of histones from chromatin or remodeling of nucleosomes. We consistently saw that the H4G94P mutants had diffuse nucleosomal ladders (Figure 7B), indicative of either rapid repositioning or random spacing of the nucleosomes within the cell population. However, if the H4G94P nucleosomes were rapidly repositioning, there should be an increased accessibility to MNase, which was not observed. Therefore, we suggest that H4G94P containing nucleosomes are not accurately spaced on the yeast genome. Even spacing of nucleosomes along the genome requires ATP-dependent nucleosome remodelers. It is noteworthy, therefore, that the terminal phenotypes of certain conditional mutants of the essential Sth1 ATPase subunit of the RSC ATP-dependent nucleosome remodeler are reminiscent of those of the H4G94P mutant [49]. Indeed, we found that the nucleosomes formed with the H4G94P mutant were remodeled less efficiently than WT H3/H4 containing nucleosomes (Figure 9A,B).

Distortions due to the G94P substitution did not prevent NCP formation *in vitro* (Figure 8), but led to NCPs that were slightly destabilized relative to WT. More importantly histone octamers were not even formed with the H4 G94P mutant. The C-terminal tail of H4 forms a parallel β sheet with H2A in the nucleosome, which is involved in the docking of H2A/H2B dimers to the H3/H4 tetrasome. This is one of several contacts between the H2A/H2B dimer and H3/H4, which cooperate to stabilize the nucleosome. SWI/SNF (‘SWItch/Sucrose Non-Fermentable’) independent (SIN) mutants of H2A and H3 [50], which are located near the DNA, and not at the interface between H4 and H2A, do not have the severity of functional or physical defects as H4^G94P^[51,52]. However, the deletion of the H4 C-terminal tail from 97 to 102, but not 100 to 102, causes lethality in yeast [53], which suggests that there is an important interaction that is altered by the H4G94P mutation. H4 Y98 lies at the H4-H2A interface and could easily be influenced by the H4G94P substitution. Indeed, Y98 substitution mutants have a range of phenotypic severity from lethality to viability [53,54], and the H4Y98H temperature sensitive mutant fails to form histone octamers [51].

The histone octamers formed with H4^G94P^ and H4^∆94^ were unstable (Figure 8A). As no similar biochemical and biophysical data have been reported for other lethal H4 substitutions, we looked for parallels between the behavior of the H4^G94P^ and H4^∆94^ nucleosomes and H2A substitutions and deletions in the H4 docking domain. In particular H2A^V101I^ destabilizes nucleosomes similarly to H4^G94P^, and suppresses a defect in the remodeler FACT [55]. Deletions of the entire H2A docking region destabilize nucleosomes and have altered electrophoretic mobility similar to that of the H4^∆94^ NCPs (Figure 8) [39]. Furthermore, this region of H2A was needed for nucleosome remodeling and mobilization of nucleosomes by RSC [39]. As we observed less efficient nucleosome remodeling for the G94P mutant relative to the WT NCP (Figure 9), remodeling must be highly sensitive to minor perturbations that occur in the interaction between the C-terminal tail of H4 and H2A. This interface has been shown in several studies to be important for many cellular functions [23,32,54,56]. Therefore, we conclude that the decreased flexibility of the H4 C-terminus hinders nucleosome remodeling, and potentially any other functions, which depend on maintenance of correctly assembled histone octamers in the cell.

## **Conclusions**

The functional consequences of altering the conformational flexibility in the C-terminal tail of H4 are severe. Despite the detrimental effects of the histone H4 G94P mutant on viability, nucleosome formation was not markedly altered *in vivo*. However, histone octamer stability and nucleosome stability as well as nucleosome sliding ability were altered *in vitro*. These studies highlight an important role for correct interactions of the histone H4 C-terminal tail within the histone octamer and suggest that maintenance of a stable histone octamer *in vivo* is an essential feature of chromatin dynamics.

## **Methods**

### **Plasmids and yeast strains**

The plasmids and yeast strains used in this study are listed in Tables 1 and 2. Yeast were grown at 30°C, in yeast peptone dextrose (YPD) or synthetic medium containing either 2% glucose (SC) or 2% galactose (Sgal) as indicated in the text [57]. Epitope tags and gene replacements were introduced into the genome by PCR mediated one-step gene replacement.

### **Site directed mutagenesis**

Mutations were introduced into plasmids, as indicated, using either the Stratagene Quick Change, or Stratagene Quick Change XL Site-Directed Mutagenesis kit according to the manufacturer’s instructions (Agilent Technologies, Santa Clara, CA, USA).

### **Construction of plasmid borne and integrated H4G94P and H4G94A yeast strains**

pEMHE81 (Table 1) [58], expressing H3 and H4 under native promoter control, was mutagenized to create the H4G94A and H4G94P mutations. Sequences of primers used for mutagenesis are available upon request. Following sequencing, the mutated H3/H4 plasmids were transformed into SNY090 derived from RMY102 to create strains SNY091, SNY092 and SNY095. To integrate the mutant and WT histones into the genome, PCR primers HHTF2-TRP-pRS414-F (TAG AGA CAT CGG ATA TAG ACA CTC CAC AAT ACA GCA TTG TCC AGA GCA GAT TGT ACT GAG), and HHTF2-TRP- pRS414-R (TGG TTG CCT CTA CAA TGG TAA TAT GTA GAC AGT GAT TAC CTT TAC G) were used to amplify the H3, H4 and *TRP1* components from these pEMHE81 based plasmids. The PCR products were introduced into W1588-4c at the *HHT1/HHF2* genetic locus. Replacement with WT H3 and H4 created strains BKD203 and BKD204; replacement with H4G94A, BKD210; and replacement with H4G94P, BKD207.

*HHT1* and *HHF1* were simultaneously deleted in W15884a with KAN amplified from pFA6a-kanMX6 using primers HHFT1-DEL-F (ATA TTT GCT TGT TGT TAC CGT TTT CTT AGA ATT AGC TAA ACG GAT CCC CGG GTT AAT TAA) and HHTF1-DEL-R (ATT GTG TTT TTG TTC GTT TTT TAC TAA AAC TGA TGA CAA TGA ATT CGA GCT CGT TTA AAC), creating strain BKD215.

### **Analysis of number of cell divisions on plates**

Asynchronous cultures were grown to log phase in synthetic medium lacking Ura and containing 2 % galactose (Sgal -Ura). Cells were counted using a hemocytometer. In all, 1,000 cells from each strain were plated onto minimal medium containing 2 % glucose but lacking uracil (SC -Ura). A subset of 100 cells were examined at 0, 4, 8, 16, and 24 h to determine the number of times the cells had divided.

### **Flow cytometry**

A total of 1 ml of log phase cells were harvested and resuspended in 300 μl of 50 mM Tris pH 7.9. Ethanol was added to a concentration of 70%, and samples were rocked for 1 h at room temperature. Cells were treated with RNase, stained with Sytox green, sonicated, and submitted for flow cytometry.

### **Growth of viable G94P mutants on glucose and 5-FOA**

RMY102 (Table 2) was transformed with pEMHE81-based plasmids containing WT, or mutant histone H4 (Table 1). Transformants were selected on Sgal -Ura -Trp. Transformants were grown in Sgal -Ura -Trp liquid culture to mid-log phase and cells were counted with a hemocytometer. Approximately 300 cells were plated onto Sgal -Ura -Trp or SC -Ura -Trp (glucose) plates. Cells were grown at 30°C until colonies were visible. Plates were imaged, and the number of colonies was counted. For the 5-FOA experiment cells were grown as above, but approximately 200 cells were plated onto Sgal -Ura -Trp plates. After colony formation, these were replica plated onto -Trp 5-FOA (0.75 g/l) plates followed by Sgal -Ura -Trp plates and grown at 30°C prior to imaging.

### **Growth curves and cell counting**

Indicated strains (MCY091, MCY094, and MCY097) were grown overnight in Sgal -Ura, diluted to an OD_600_ of 0.3, and grown an additional 4 to 5 h to an OD_600_ of 0.5 to 0.7. Cultures were again diluted to an OD_600_ of 0.2 and glucose was added to 2%. The OD_600_ of each culture was measured at 0, 1, 2, 3, 4, 5, 6, 8, and 10 h after the glucose shift. Cells were maintained in logarithmic growth as described below. Cell counts for MCY091, MCY094, and MCY097 were calculated in parallel with the OD measurements using a hemocytometer.

### **Budding morphology and DAPI staining**

Asynchronous, mid-log phase cultures (MCY091, MYC094 and MCY097) grown in Sgal -Ura, were diluted into either Sgal -Ura or SC -Ura at a density of 0.2 OD_600_/ml. Growth medium and cells were removed and replenished with fresh medium as needed to keep the cells at a constant volume and at an OD_600_ < 1.0 for the duration of the experiment. Cells were fixed with 70% EtOH and stained with DAPI following standard procedures [57].

### **Protein expression and purification**

Plasmid T60 H4^G94P^ was generated using site-directed mutagenesis from the plasmid pST39T60Xtal. The expression and purification of the protein complex were carried out as previously described [17].

Full-length yeast Asf1 was purified as described previously [18]. Full-length yeast Asf1 (residues 1 to 279) was labeled at position −1 with Alexa Fluor 532 (Invitrogen; Life Technologies, Grand Island, NY, USA) according to manufacture recommended protocols, yielding yAsf1*^532^.

The expression and inclusion body preparation of *Xenopus laevis* histones H2A, H2B, H3 and H4 with amino acid residue substitutions H3 C110A and H4 T71C, were as previously reported [37] with modifications [18]. The denatured histones were then passed through Q Sepharose Fast Flow resin (GE Healthcare, Piscataway, NJ USA) to remove DNA and acidic contaminants. The flow through was then bound to SP Sepharose Fast Flow resin (GE Healthcare) and eluted by increasing the concentration of NaCl. Fractions containing the pure protein were dialyzed into water and lyophilized. Histone H3 with an amino acid substitution of H3 C110A was used to avoid any unwanted cysteine/dye reaction [41]. Histone H4^G94P^ and H4 Δ94-102 (H4^∆94^) were from the plasmid pET3a H4T71C. Histone H4 with a mutation of T71C was used to label H4 without interfering with the H3/H4 dimerization interface [41]. H4 was labeled according to manufacturer recommended protocols and then assembled into H3/H4 tetramers [37] with histone H3 to produce H3/H4 tetramers, H3/H4^G94P^ tetramers, and H3/H4^∆94^ tetramers.

### **Preparation of DNA**

80 bp DNA fragments of the *Xenopus laevis* 5 S rDNA gene sequence and a 146 bp DNA fragment of the strong nucleosome positioning sequence known as the 601 sequence [38,59] were prepared and purified as previously described [18]. A 208 bp DNA fragment consisting of the 601 sequence at one end, 0-N-63, was generated using PCR with the primers: 601_208_F 5′-CA GGA TGT ATA TAT CTG ACA CGT GCC TGG and 601_208_R 5′-GGA AAG CAT GAT TCT TCA CAC CGA GTT C, as in [44]. The PCR product was purified in the same way as the 146 bp 601 DNA.

### **Crystallization, structure determination and analysis**

Crystallization conditions for Asf1-H3/H4^G94P^ were identified in screens using the JCSG Core I Suite screen (Qiagen, Valencia, CA USA) and were optimized. Hexagonal prism crystals of 150 × 100 × 100 Å formed in 0.2 M Mg acetate and 7.14% PEG 3350 after 2 days at 25°C. Crystals were cryoprotected with a solution of 0.2 M Mg acetate, 20% PEG 3350, and 10% glycerol prior to flash freezing in liquid nitrogen.

Diffraction data were collected on the Molecular Biology Consortium Beamline 4.4.2 (MB-CAT) at the Advance Light Source, Lawrence Berkeley National Laboratories. The crystals formed in space group P3_1_21 with cell dimensions a = b = 97.69 c = 115.07. Data were processed using d*trek and the structure was solved by molecular replacement using Asf1-H3/H4 structure (PDB:2HUE) [23] and PHASER [60,61]. The model was built using coot and refined using Refmac (CCP4) [62], with group TLS refinement included in the final round. The refined model contains residues 1 to 156, 159 to 164 of Asf1 (chain A), 60 to 135 of H3 (chain B), and 20 to 102 of H4 (chain C). Asf1 residues 157 to 158 were too disordered to include in the model. The structure has PDB ID 4EO5. Structure diagrams were made using Pymol [63]. RMSD calculations of the structures were calculated using SwissPDBviewer.

### **Fluorescence assays**

All thermodynamic measurements were carried out at 20°C in buffer that contained 150 mM KCl, 2 mM MgCl_2_, 10 mM Tris–HCl pH 7.5,1% glycerol, 0.05% Brij-35, and 0.5 mM tris(2-carboxyethyl)phosphine (TCEP). Unlabeled H3/H4 or H3/H4^G94P^ was titrated into a cuvette containing 1 nM yAsf1*^532^ and the decrease of yAsf1*^532^ fluorescence was monitored. Double measurements were made with an integration time of 0.5 s on a Horiba Fluorolog-3 spectrometer, using a 0.5 cm path-length cuvette. The excitation wavelength was 528 nm, with a slit width of 5 mm; emission was recorded at 548 nm with a slit width of 7 mm. The buffer was scanned in the same range used in all experiments for background contributions to the readings and was corrected for in each spectrum. Varying incubation times (0 to 30 minutes) confirmed that the fluorescence signal had reached equilibrium by 10 minutes. The reactions were allowed to equilibrate at 20°C for at least 10 minutes prior to measurement, and a minimum of three independent experiments were performed for each sample. Control samples in which buffer was added to the cuvette instead of histones discounted the effect of buffer on the quenched Asf1 signal. A ligand-depleted binding equation was used to fit the fluorescent quenching data (Equation 1).

(1) $$\begin{array}{l} {\text{F}}_{\text{i}}=\;1+\left(\text{Fmax}\right)*((\left({\text{K}}_{\text{D}}+\left[\text{yAsf}1*\right]+{\left[\text{H3H}4\right]}_{\text{i}}\right)-\text{sqrt}(\left({\left({\text{K}}_{\text{D}}+\left[\text{yAsf}1*\right]+{\left[\text{H3H}4\right]}_{\text{i}}\right)}^{2}\right)-\\ \left(4*\left[\text{yAsf}1*\right]*\left[\text{H3H}4\right]\text{i}\right)))/2*\left[\text{yAsf}1\right]))) \end{array}$$

Where i indicates the varying concentrations of H3/H4 that were titrated into the yAsf1*.

### **Coimmunoprecipitation of Asf1 and histone H4 mutants**

The 350 ml cultures were grown to an OD_600_ of 1.0. Cells were harvested, washed and resuspended in phosphate-buffered saline (PBS) containing 4.5 mM of the crosslinker DSP (Pierce 22585; Pierce, Rockford, IL USA) dissolved in dimethylsulfoxide. After 30 minutes, crosslinking reactions (RT) were quenched with 20 mM Tris, pH 7.5 and allowed to incubate for 15 minutes. Cell pellets were lysed with glass beads in 40 mM 4-(2-hydroxyethyl)-1-piperazine-ethanesulfonic acid (HEPES) pH 7.5, 1 mM dithiothreitol (DTT) containing Roche Complete ethylenediaminetetraacetic acid (EDTA)-free protease inhibitor cocktail (Roche Indianapolis, IN USA) [64]. Lysates were cleared in a tabletop centrifuge for 10 minutes at 12,000 *g,* and 4°C for 10 minutes. Cleared lysates were subjected to ultracentrifugation (SW55-Ti Rotor; Beckman) at 149,000 *g* for 1 h at 4°C. Protein concentration was determined by Bradford. Then, 50 μl of EZ-view Anti-Myc beads (Sigma, St. Louis, MO USA) were added to 10 to 15 mg protein and incubated overnight at 4°C. Beads were washed four times with lysis buffer containing 0.1% NP-40. Proteins were eluted by boiling in 1 × SDS sample buffer.

Equal amounts of eluate were loaded onto a 15% SDS-PAGE gel and transferred to nitrocellulose (400 mA, 15 minutes). Membranes were blocked with Tris-buffered saline/Tween (TBST) + 5% bovine serum albumin (BSA) for 1 h and probed overnight at 4°C with either a 1:1,000 dilution of anti-c-Myc antibody (Santa Cruz, sc-789, Santa Cruz Santa Cruz, CA USA), 1:1,000 dilution of anti-H3 antibody (Abcam, 1791; Abcam Cambridge, MA USA), or a 1:500 dilution of anti-H4K12ac antibody (Upstate, 06-761MN, Upstate Billerica, MA, USA). Detection was performed after probing with a 1:5,000 dilution of anti-rabbit Alexa Fluor 680 (Invitrogen, A21100) by visualizing on an Odyssey infrared imaging system (LI-COR Biosciences). Electrochemiluminescence (ECL) detection was performed after probing with a 1:25,000 anti-rabbit horseradish peroxidase (HRP) (Sigma A-1949) and 1:80,000 anti-rat HRP (Sigma A5795).

### **Chromatin fractionation**

Samples were processed as previously described [65]. Briefly, 2 × 10^8^ asynchronous cells were harvested and washed before spheroplasting with Zymolyase 100 T at room temperature. Spheroplasts were washed twice with wash buffer containing fresh protease inhibitors (Roche mini complete EDTA free). Cells were resuspended in Lysis buffer, DNA concentration was determined, and the same number of DNA equivalents from each strain was fractionated by centrifugation to separate the supernatant (soluble protein faction) from the pellet (chromatin fraction). Pellets were resuspended in 1 × SDS sample buffer and loaded onto a 4% to 20% gradient SDS-PAGE. Membranes were stained with amido black and blocked with TBST + 5% BSA for 1 h and then probed overnight at 4°C with the following 1˚ antibody dilutions: 1:1,000 anti-H3 (Abcam, 1791), 1:1,000 anti-H4K12ac (Upstate, 06-761MN), and 1:500 anti-tubulin (Serotech NCMCA785). Detection was performed after probing with a 1:25,000 anti-rabbit HRP (Sigma A-1949) and 1:80,000 anti-rat HRP (Sigma A5795) using Immobilon Western Chemiluminescent HRP Substrate (catalog no. WBKLS0050).

### **Chromatin immunoprecipitation and real time PCR analysis**

ChIP and real time PCR analysis were performed as previously described [65]. Briefly, 1.25 × 10^8^ cells were treated with formaldehyde, washed, and stored at −80˚ until use. Cells were lysed at 4°C with glass beads in Lysis buffer containing a protease inhibitor cocktail (Calbiochem/Novagen Cocktail set IV, catalog no. 539136)). Chromatin was shared by sonication to approximately 500 bp fragments. A total of 50 μl of supernatant was saved for input controls and was mixed with 30 μl ChIP elution buffer before storing overnight at 4°C. The remaining sample was used for the immunoprecipitation and added to 20 μl Dynabeads Protein A (Invitrogen catalog no. 100-02D) and incubated overnight at 4°C. Beads were washed and collected. Both protein of interest (IP) and genomic DNA (Input) samples were resuspended in 80 μl of ChIP Elution buffer and 20 μl of Pronase (20 mg/ml) and incubated in a PCR cycler using the following program (2 h, 42°C; 8 h, 65°C; hold, 4°C) to reverse DNA-protein crosslinks and digest proteins. The DNA was purified using the MiniElute DNA Purification kit from Qiagen (catalog no. 28004).

ChIP quantification was performed via real-time PCR with a Roche Applied Sciences LightCycler 480 and the LightCycler 480 SYBR Green I Master Mix kit (Roche catalog no. 04707516001). Each ChIP sample was analyzed in triplicate using the following program: 10 minutes denaturation, 95°C; 10 s, 95°C; 15 s, 54°C; 15 s, 72°C amplification 50 times, 5 s, 95°C; 1 minute, 65°C, increase at 2.5°C/s until 97°C, acquiring data 7 times per °C, and finally cooling 4°C.

Standard curves were generated using Input DNA dilutions of 1:10, 1:100, 1:1,000 and 1:10,000. The dilutions that gave Ct values within the linear range were then used for each ChIP sample (IP and Input) and analyzed in triplicate. The melting curve analysis for each primer pair indicated the generation of a single PCR product. IP samples were normalized to Input samples using the formula (1/2^(IP CT - Input CT).

The primers used were: RDN5 F: GCGAAATGCGATACG TAATGTG; RDN5 R: GGCGCAATGTGCGT TCA; NTS2 F: CGGATGCGGGCGATAAT; NTS2 R: GCCGACATTCTGTCCCACAT; TEL7L-XC-RTF: TCA GTACTAAATGCACCCACATCA; TEL7L-XC-RT R: TG GGTAATGGCACAGGGTATAG; TEL7L-XR-RT F: AACCACCATCCATCCATCTCTCTACTT; TEL7L-XR-RT R: AGAACAACAGTACAGTGAGTAGGACA; TEL5R-XC-RT F: CCATGGAGTGGAATGTGAGAGTAG; TEL5R-XC-RT R: TGCCATACTCACCCTCACTTGTT; TEL5 R-XR-RT F: TGGAGTTGGATATGGGTAATTGG; TE L 5R-XR-RT R: CATCCATCCCTCTACTTCCTACCA; TE L5R-YP-RT F: CGTTTGTTGAAGACGAACCAGAT: TE L5R-YP-RT R: TGTAGACCATCACGTGGTTTGTT; AC T1-RT-F: TCGTTCCAATTTACGCGTGTT; ACT1-RT-R: **CGGCCAAATCGATTCACAA;** HML-α-RT F: TCAATATTATTCGACCACTCAAGAAAG; HML-α-RT R: CG CTATCCTGTGAATTTGGATTT.

### **Immunoblotting**

Whole cell lysates were made by boiling cells in Laemmli buffer and loading equal DNA equivalents (10 μg DNA) onto SDS-PAGE gels. Antibodies and their dilutions were as follows: 1:500 anti-c-Myc (Santa Cruz sc-789), 1:50,000 anti-rabbit HRP (Sigma A-1949), 1:1,000 anti-histone H3 (Abcam ab1791), 1:500 anti-acetyl histone H4 (Lys 12) (Cell Signaling, Boston, MA USA), 1:1,000 anti-FLAG M2 (Sigma F1894), 1:1,000 anti-glyceraldehyde 3-phosphate dehydrogenase (GAPDH) (Sigma A-9521), 1:500 anti-tubulin (Serotech NCMCA785), 1:1,000 anti-acetyl-histone H3 (Lys 56) (Millipore-Upstate 07–677), 1:80,000 anti-rat HRP (Sigma A5795), 1:10,000 anti-mouse HRP (Promega W40213). Detection kits used were ECL (GE Healthcare RPN2106) or ECL Plus (RPN 2132).

### **MNase analysis**

Log phase cells were grown in SC -Ura and harvested at 0 and 8.5 h after the addition of glucose, as described under Growth Curves. Cells were fixed in 2% formaldehyde for 30 minutes at room temperature before adding glycine to 125 mM. Cells were spheroplasted with Zymolyase 100 T (10 mg/ml) in buffer Z (1 M sorbitol, 50 mM Tris pH 7.4, 10 mM β-mercaptoethanol (β-ME)) for 45 minutes. More than 90% cell wall digestion was confirmed visually by light microscopy. Three separate aliquots of spheroplasted cells were used to isolate and quantify the average amount of genomic DNA per sample as previously described [65]. A total of 0.8 μg DNA was digested with 1 U of MNase (Worthington) in 1 ml of NP buffer (1 M sorbitol, 50 mM, 5 mM MgCl_2,_ 5 mM CaCl_2_, 0.075% NP-40, 1 mM β-ME, 500 mM spermidine) at 37°C. At 0, 0.5, 1, 2, 3, 5, 10, and 15 minutes after the addition of MNase 100 μl of sample were collected and added to STOP buffer (10% SDS, 0.5 M EDTA, 0.1 mg/ml proteinase K). Samples were incubated overnight at 65°C. DNA was purified by phenol:chloroform extraction followed by EtOH precipitation. DNA recovered from each sample was resuspended in TE buffer and analyzed on a 1.2% agarose gel. DNA was visualized with ethidium bromide.

### **Electrophoretic mobility shift assays**

Electrophoretic mobility shift assays (EMSAs) were performed as previously described [18]. Mixtures of yAsf1 and H3/H4 or H3/H4^G94P^ or H3/H4^∆94^ and DNA were prepared in assembly buffer (10 mM Tris–HCl, pH 7.5, 0.5 mM TCEP). The samples were incubated at 20°C for 1 h prior to electrophoresis. A 7 % polyacrylamide gel (59:1 acrylamide:bis-acrylamide) containing 0.2 × Tris/Borate/EDTA (TBE) was pre-run for 60 minutes in 0.2 × TBE at 4°C at 70 V. The reactions were electrophoresed at 70 V for 2 to 3 h at 4°C. Bands were visualized by recording the SYBR Green I nucleic acid stain (Invitrogen) fluorescence and the Alexa Fluor 532 signal of yAsf*^532^, (if labeled Asf1 was used in the experiment) with a Typhoon 9400 Variable mode imager (GE Healthcare). The bands were quantitated using ImageQuant software.

### **Size-exclusion chromatography**

WT octamers were prepared using all WT full-length proteins (H2A, H2B, H3, and H4) as previously reported [37]. H4^G94P^ and H4^∆94^ octamers were prepared similarly. A Superdex 200 16/60 (GE Healthcare) size exclusion column was used for chromatography.

### **Reconstitution of nucleosome core particles**

Nucleosome core particles (NCP) were prepared by two different methods. One set of NCPs was formed by a continuous salt dialysis method [66]. Salt dialysis WT NCPs were formed in two ways: purified histone octamer was combined with DNA in a 0.9:1.0 octamer to DNA ratio, or purified H3/H4 tetramers, H2A/H2B dimers and DNA were combined in a 0.9:1.8:1.0 ratio. H4^G94P^ octamers were assembled by addition of H3/H4^G94P^ tetramers, H2A/H2B dimers and DNA combined in a 0.9:1.8:1.0 ratio. H4^∆94^ NCPs were prepared the same as H4^G94P^ NCPs with the exception of the use of H3/H4^∆94^ tetramers. NCPs were also prepared by microscale reconstitution [37]. Briefly, H3/H4 tetramers and H2A/H2B dimers were added to 1 μg of 601 146 bp DNA in a 1:2:1 ratio in 2 M NaCl. Then, 10 mM Tris–HCl pH 7.5 was added at specific intervals until a final NaCl concentration of 200 mM was reached.

### **Nucleosome sliding assays**

Nucleosome sliding assays were performed similarly to [44]. Drosophila dACF was kindly provided by Dr Jim Kadonaga [67], and Chd1 by Dr Gregory Bowman [44]. Briefly, 12 nM NCP in buffer 1 (20 mM HEPES, pH 7.6, 50 mM KCl, 5 mM MgCl2, 5% sucrose, 0.1 mg/ml BSA, and 1 mM DTT) was incubated with Chd1 protein and 2.5 mM ATP for the stated time points. After the noted incubation time, the reactions were quenched by the addition of 1 μg of stop DNA (supercoiled 5 S rDNA) and an equal amount of stop buffer (buffer 1 + 25 mM EDTA), and placed on ice. Reactions were then loaded onto a 7% (59:1 acrylamide:bis) gel and electrophoresed for 3 h at 70 V on ice. Gels were then stained with SYBR green I (Invitrogen) nucleic acid stain, and imaged on a Typhoon 9400 variable mode imager (GE Healthcare). Reactions containing dACF were performed similarly with some exceptions; 12 nM NCP in buffer 1 was incubated with 5 nM dACF + 2.5 mM ATP for 30 minutes, after which Chd1 was added only to the reactions containing both proteins. The reactions were incubated at room temperature for 120 minutes then quenched, stained and imaged as above.

## **Competing interests**

The authors declare that they have no competing interests.

## **Authors’ contributions**

MC and BKD carried out the molecular genetic studies and assisted in drafting the manuscript. JKS carried out the structural and biophysical studies and assisted in drafting the manuscript. SN prepared mutants and conducted coimmunoprecipitation experiments. MEAC and JKT conceived of the study, and participated in its design and coordination and helped to draft the manuscript. All authors read and approved the final manuscript.

## Supplementary Material

Additional file 1 Title: Supplemental materials. Description: File contains a Table (S1) and Figures with Legends (Figures S1-S8). Click here for file
