# Supplementary material for: The conformational flexibility of the C-terminus of histone H4 promotes histone octamer and nucleosome stability and yeast viability
Source: Epigenetics Chromatin. 2012 Apr 27;5:5. doi: 10.1186/1756-8935-5-5 (PMC3439350; doi:10.1186/1756-8935-5-5)
Supplement: Additional file 1 — Title: Supplemental materials. Description: File contains a Table (S1) and Figures with Legends (Figures S1-S8). [file 1756-8935-5-5-S1.docx]

**Table S1. Structural comparison of Asf1-H3/H4 and Asf1-H3/H4^G94P^**

| **Regions compared** | **rmsd backbone (Å)** |
| --- | --- |
| **Asf1-H3/H4** | 1.06 |
| **Asf1** | 0.94 |
| **Asf1 1-145** | 0.47 |
| **Asf1 146-164** | 1.58 |
| **H3** | 0.46 |
| **H4** | 0.46 |
| **H4 20-78** | 0.29 |
| **H4 79-101** | 1.01 |
| **H4 92-101** | 1.35 |


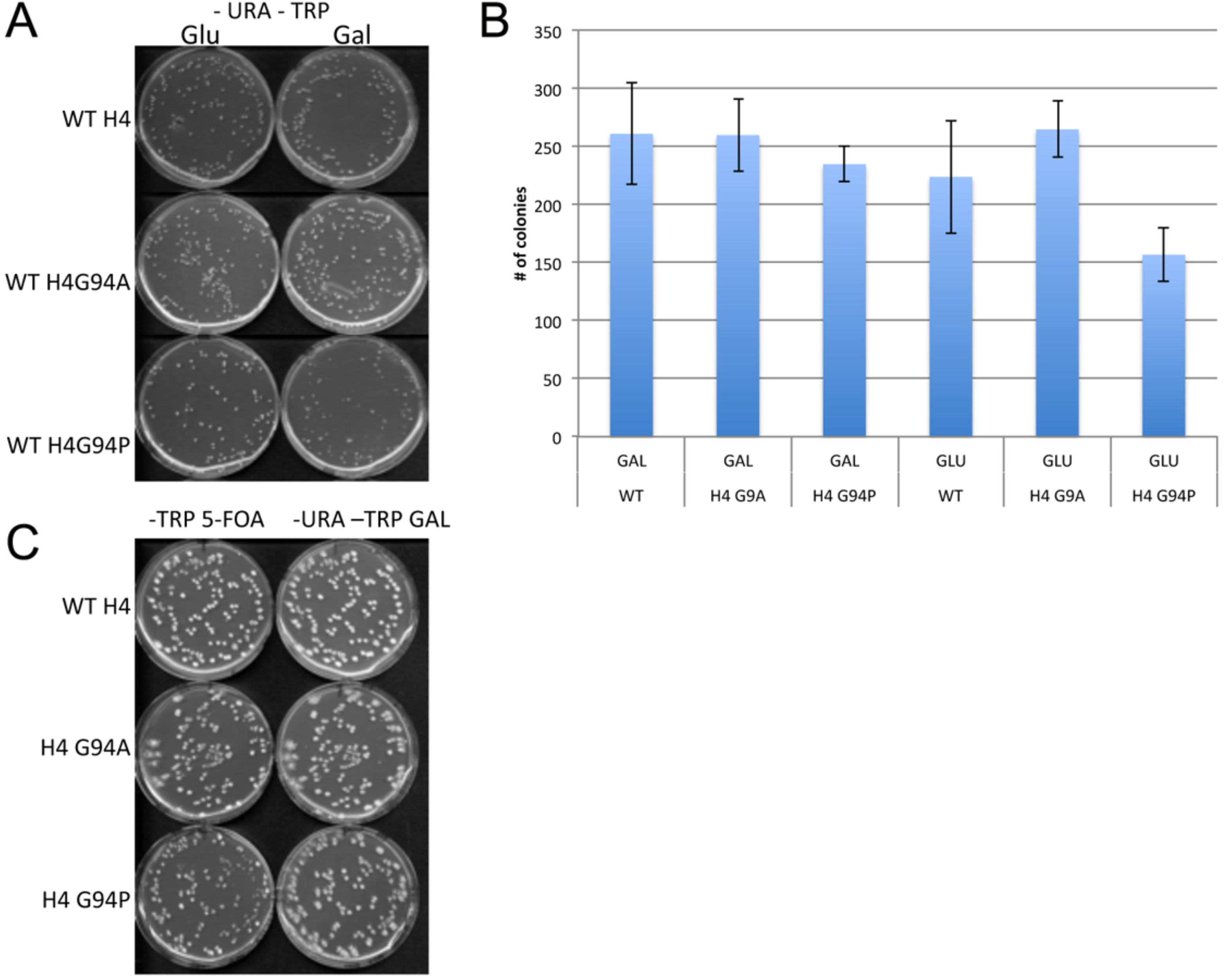


**Figure S1. Analysis of colony formation due to the G94 mutations upon transcriptional repression of the wild type histones.**  (A) RMY102 which contains pRM102 (*CEN4 ARS1 p(GAL10)-HHT2 p(GAL1)-HHF2 URA3)* was transformed with pEMHE81 (*CEN6 ARSH4 HHT2* and *HHF2* *TRP1*) based plasmids containing WT, or mutant histone H4. Transformants were selected on Sgal -Ura -Trp. Transformants were grown in Sgal -Ura –Trp liquid culture to mid-log phase and cells were counted with a hemocytomter. ~300 cells were plated onto Sgal –Ura -Trp or SC -Ura -Trp (glucose) plates. Cells were grown at 30˚C until colonies were visible (WT and G94A on glucose, 2-3 days; G94P mutants on glucose, and all strains on galactose, 3-4 days.) (B) As in (A). Colony counts for three independent transformants plated in triplicate for each growth condition (9 plates per data point). Bars represent the standard deviation of the mean. 70% of the G94P mutant cells were able to form colonies after WT histone expression was repressed with glucose. (C) Cells were grown as in A, but ~200 cells were plated onto Sgal -Ura -Trp plates. After colony formation, these were replica plated onto -Trp 5-FOA (.75g/L) plates followed by Sgal –Ura –Trp plates. Cells that are phenotypically URA+ because they cannot lose the pRM012 plasmid will die on 5-FOA, whereas those that have lost the plasmid, and are phenotypically ura-, will survive. All of the G94P mutant colonies, although slow growing, were able to grow on 5-FOA following replica plating.

**
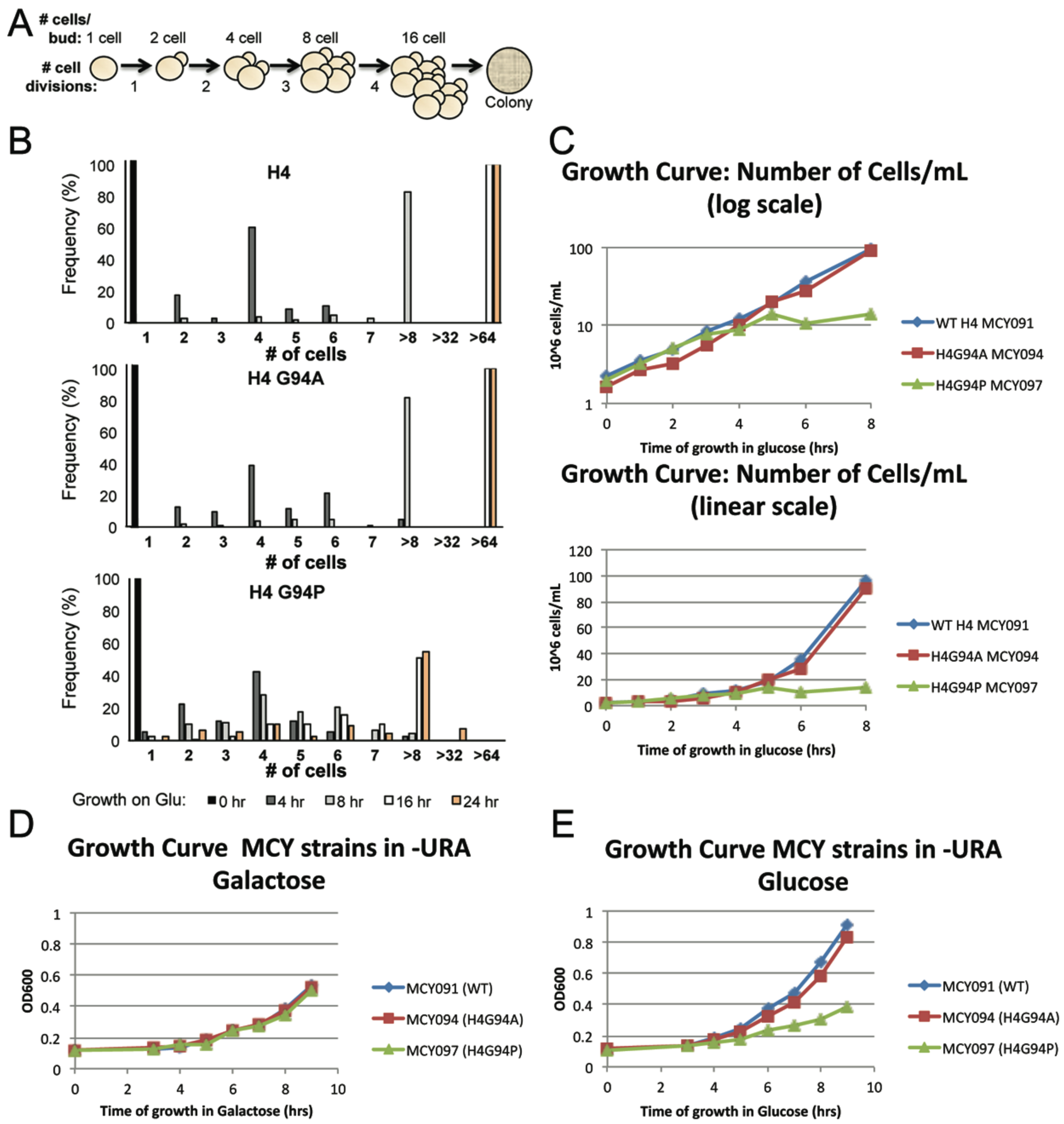
Figure S2. Cell division stops after repressing expression of the wild type H4 when H4G94P is integrated into the genome.**  (A) Schematic for the analysis of cell division after repressing WT histone expression. (B) H4G94P mutants divide no more than 3 times in the presence of glucose. The single cells from the strains used in (Figure 3B) were plated onto glucose plates and the number of cells derived from a single cell at the indicated time points was plotted. (C). Number of cells/ml of strains MCY091 (H4), MCY094 (H4G94A), and MYC097 (H4G94P) at the indicated times after transfer to glucose media to repress wild type H4 expression (shown with log and linear y-axis scales). (D) Growth curve for the same strains used in (A) in galactose containing media. (E) Growth curve for the same strains used in (C) in glucose containing media. All cells were diluted to an OD_600_ of 0.1 at the beginning of the growth analyses.

**
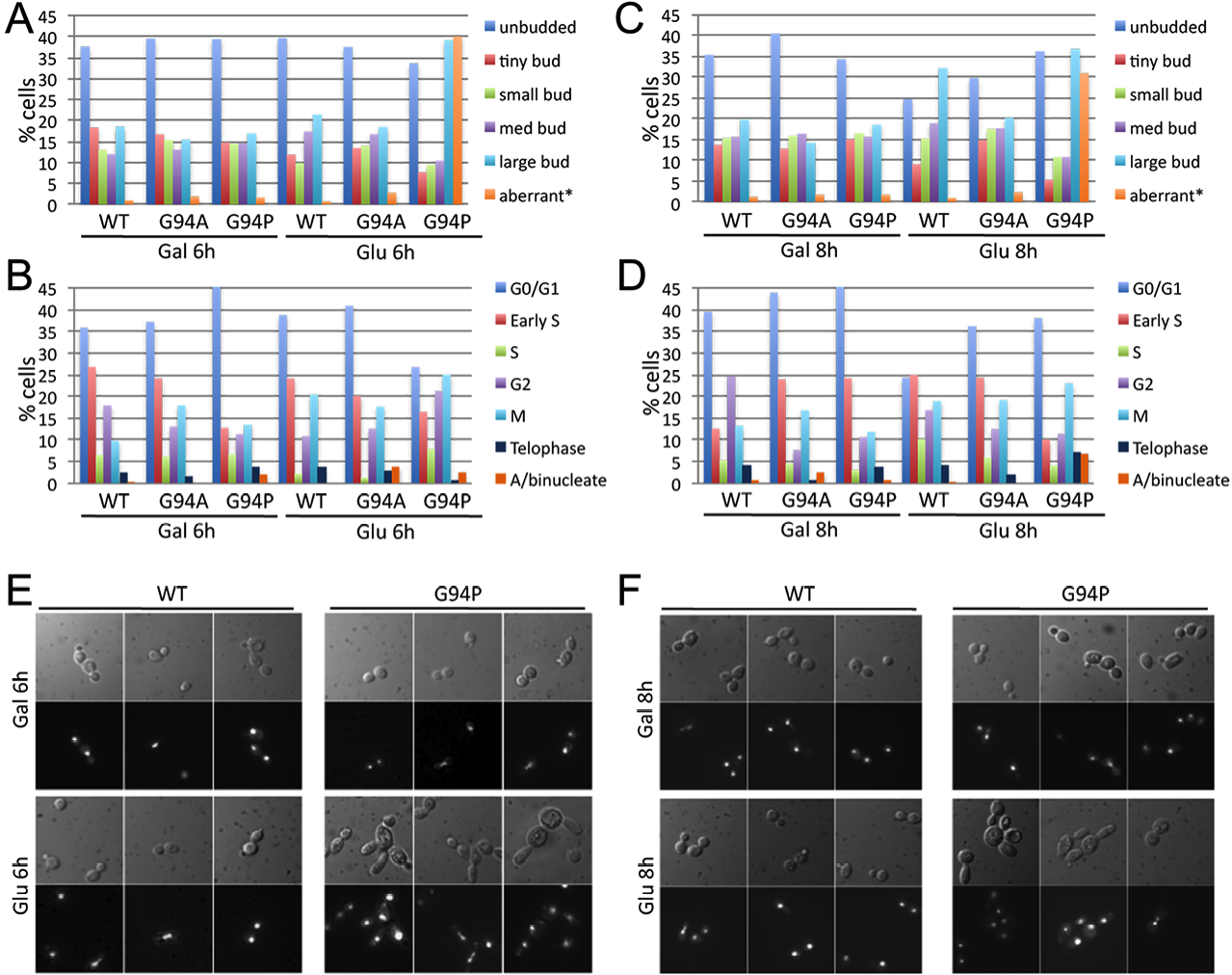
**

**Figure S3. Analysis of the effect of limiting conformational flexibility of the H4 C-terminal tail on the yeast cell cycle. (**A -F) cells were grown in S-gal-Ura medium as described in the Materials and Methods. (A, B and C) 6 hr after growing cells in galactose or 6 hr after repressing WT H4 expression with glucose. (D, E and F) as in A-C, but at 8 hr. (A) After 6 hours, H4G94P mutants grown in glucose have an increase in the percentage of cells with large buds at the expense of cells with tiny, small and medium buds compared to WT or H4G94A mutant cells. (D) After 8 hours, H4G94P mutants grown in glucose have an increase in the percentage of unbudded and large budded cells at the expense of cells with tiny, small and medium buds compared to WT or H4G94A mutant cells. 500-600 cells of each strain were scored for each condition: MCY091, WT; MCY094 H4G94A; MCY097, H4G94P. The aberrant category is expressed as a percentage of all cells counted for each strain. These aberrant cells were of three main types: those that were abnormally large regardless of bud size, those that were very large, round and unbudded, and those with elongated buds or bud-like projections. (B) 6 hr after repressing WT histone expression, H4G94P mutants do not accumulate at any one phase of the cell cycle. Approximately 150 cells for each strain were scored for each condition. The nuclear cell division cycle was defined as follows: G0/G1 - unbudded or large budded cells with central nuclei. Early S - cells with a tiny bud. S - cells with small, medium or large buds with mothers containing a single nucleus that had not migrated to the mother-bud neck. G2 to M transition - cells with nuclei positioned at the mother-bud neck. M - cells with a contiguous DAPI signal shared between the mother and bud. Telophase - cells with nuclei at opposite poles. Anucleate - a single large cell with no DNA staining, or a daughter cell with DNA staining attached to a mother cell without staining. (E) As in B, but at 8 hr. (C) Morphological changes 6 hr after repressing WT H4 expression in the H4G94P mutants. DAPI and DIC images are shown for the same cells. (F) As in E, but at 8 hr.

**
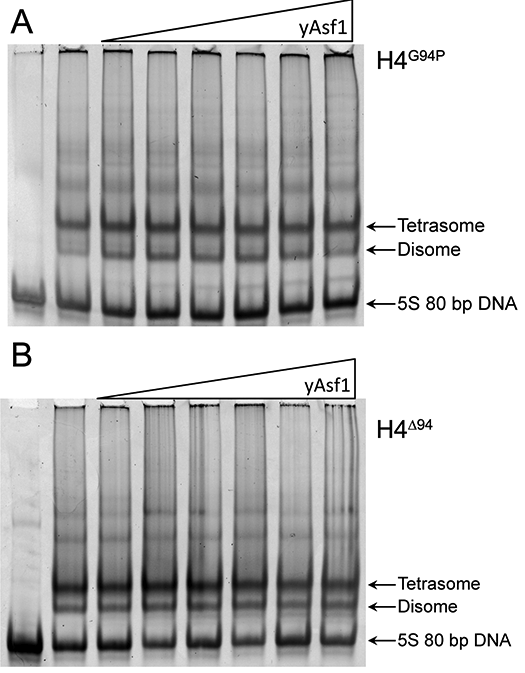
**

**Figure S4. Asf1 is unable to dissociate H3/H4^G94P^ and H3/H4^Δ94^ tetramers from DNA.** 0.8 μM H3/H4^G94P^ or H3/H4^Δ94^ histones were incubated with 0.4 μM 5S DNA for 30 minutes at 20°C. Unlabeled yAsf1 was then added at increasing concentrations (0.0, 0.2, 0.4, 0.6, 0.8, 1.0, 2.0 μM) and the reactions incubated for 1 hour. The samples were then analyzed by native PAGE, stained with SYBR Green I nucleic acid stain (Invitrogen), and imaged on a Typhoon 9400 variable mode imager. The first lane contains DNA without histones or Asf1, the second lane contains DNA and H3/H4 with no Asf1, and the remaining lanes contain DNA and H3/H4 with increasing concentrations of Asf1. There is no decrease or disappearance of tetrasomes or disomes formed with H3/H4^G94P^ (A) or H3/H4^Δ94^ (B) with the addition of yAsf1.

**
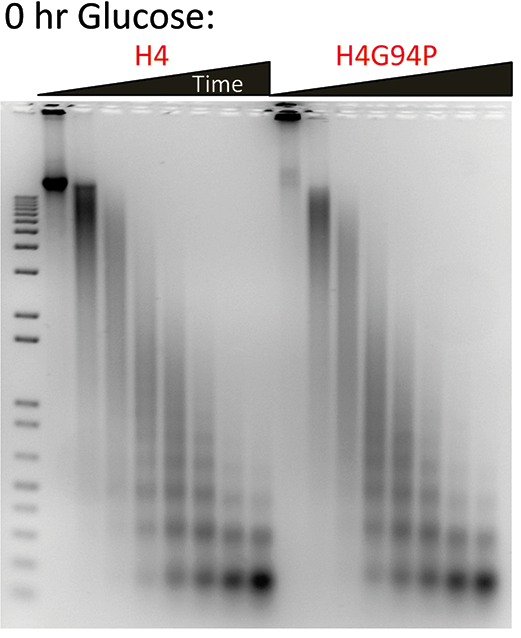
**

**Figure S5. Microccocal nuclease analysis showing no difference at time 0 between WT and G94P.** Samples from the same experiment shown in Fig. 6B were analyzed at time 0, immediately after adding glucose.

**
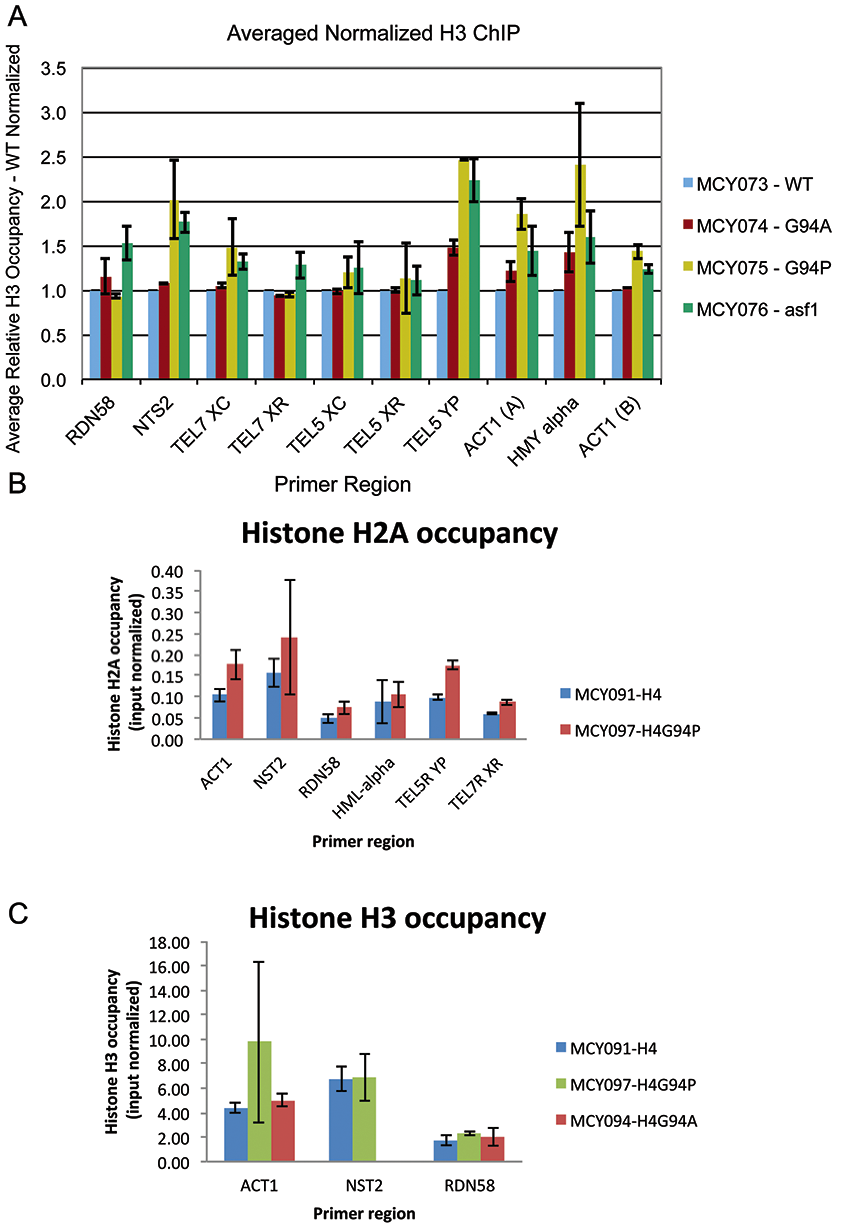
**

**Figure S6. Histone H3 occupancy on the genome as measured by ChIP is not significantly altered in the H4G94P mutant.**  Shown are the average and standard deviation of three independent experiments performed in the indicated strains. WT H4 expression was repressed for 8 hours prior to ChIP. (A) Strains (RMY102 derivatives) that constitutively express histone H4 as indicated. (B) and (C) Strains that required WT H4 to be repressed by the addition of glucose.

**
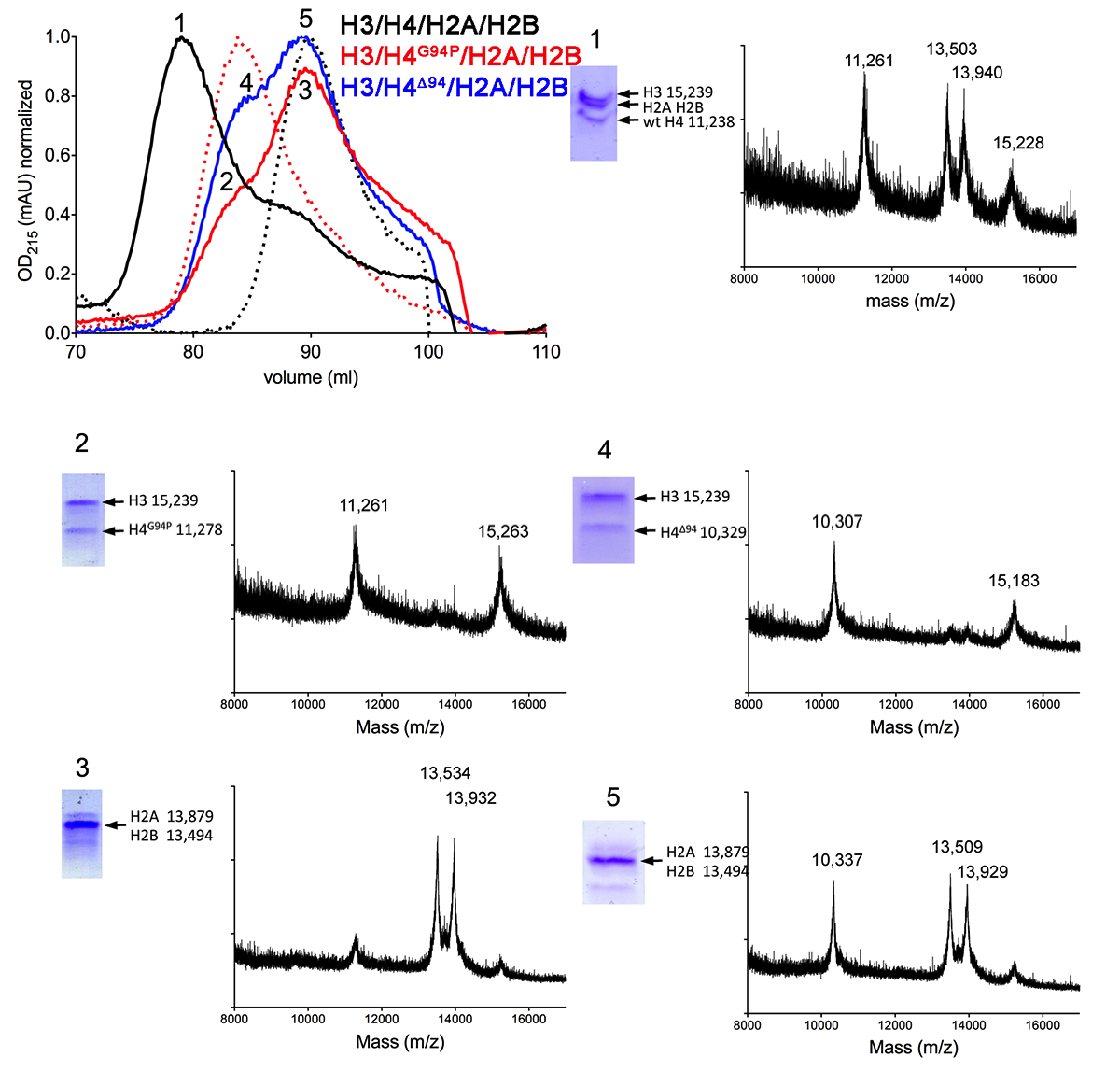
**

**Figure S7. Presence of histone proteins in octamer, tetramer, and dimer species.** Fractions from size exclusion chromatography of wild type and mutant histones prepared as octamers were run on 18% SDS-PAGE. MALDI-TOF was then conducted on the fractions. The band and spectrum of representative fractions is shown for octamer composed of wild type H4 (1), H3/H4^G94P^ (2) and (3), H3/H4^Δ94^ (4) and (5).

**
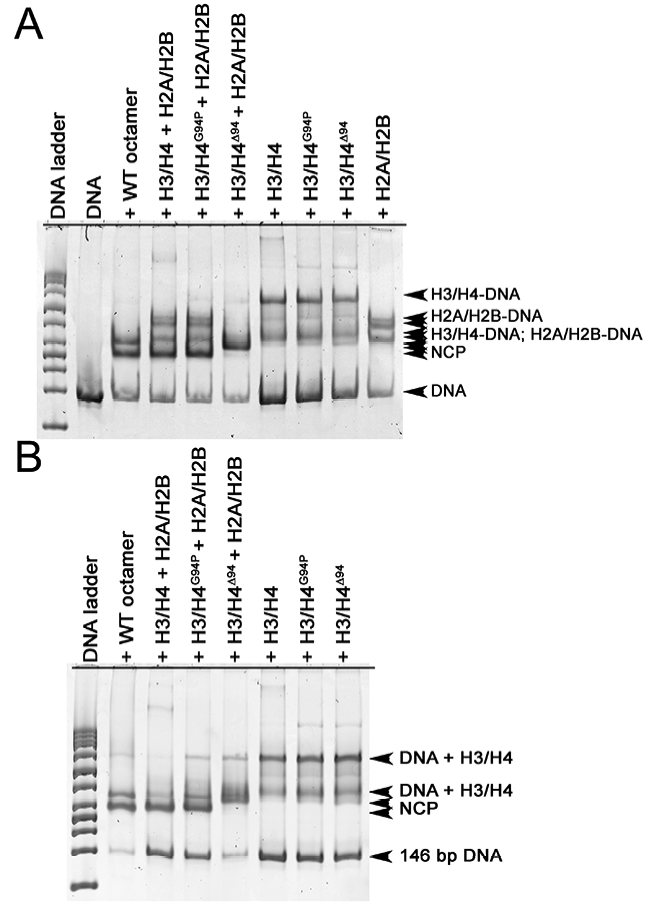
**

**Figure S8. Electrophoretic Analysis of Histone-DNA Species.**  **(A)** H4^G94P^ and H4^∆94^ form nucleosome core particles (NCPs) using microscale reconstitution (DNA + H3/H4 + H2A/H2B) procedures. Lanes: 1) 50 bp DNA ladder, 2) 146 bp 601 DNA, 3) NCPs formed with octamer and DNA, 4) NCPs formed with H3/H4 tetramers, H2A/H2B dimers and DNA, 5) NCPs formed with H3/H4^G94P^ tetramers, H2A/H2B dimers and DNA, 6) NCPs formed with H3/H4^Δ94^ tetramers, H2A/H2B dimers and DNA, 7) DNA + H3/H4, 8) DNA + H3/H4^G94P^, 9) DNA + H3/H4^Δ94^ 10) DNA with H2A/H2B. **(B)** Lanes: 1) 50 bp DNA ladder, 2) NCPs formed with octamer and DNA, 3) NCPs formed with H3/H4 tetramers, H2A/H2B dimers and DNA, 4) NCPs formed with H3/H4^G94P^ tetramers, H2A/H2B dimers and DNA, 5) NCPs formed with H3/H4^Δ94^ tetramers, H2A/H2B dimers and DNA, 6) DNA + H3/H4, 7) DNA + H3/H4^G94P^, 8) DNA + H3/H4^Δ94^.

Arrows indicate the positions of the NCPs, H2A/H32B-DNA complexes, tetrasomes, and free DNA.
